# Formal Verification of Real-Time Autonomous Robots: An Interdisciplinary Approach

**DOI:** 10.3389/frobt.2022.791757

**Published:** 2022-04-13

**Authors:** Mohammed Foughali, Alexander Zuepke

**Affiliations:** ^1^ Université Paris Cité, IRIF, CNRS, Paris, France; ^2^ Chair of Cyber-Physical Systems in Production Engineering, Technical University of Munich, Garching, Germany

**Keywords:** robotics, real-time systems, formal methods, timed automata, locking protocols

## Abstract

Due to the severe consequences of their possible failure, robotic systems must be rigorously verified as to guarantee that their behavior is correct and safe. Such verification, carried out on a *model*, needs to cover various behavioral properties (e.g., safety and liveness), but also, given the timing constraints of robotic missions, real-time properties (e.g., schedulability and bounded response). In addition, in order to obtain valid and useful verification results, the model must faithfully represent the underlying robotic system and should therefore take into account all possible behaviors of the robotic software under the actual hardware and OS constraints (e.g., the scheduling policy and the number of cores). These requirements put the rigorous verification of robotic systems at the intersection of at least three communities: the robotic community, the formal methods community, and the real-time systems community. Verifying robotic systems is thus a complex, interdisciplinary task that involves a number of disciplines/techniques (e.g., model checking, schedulability analysis, component-based design) and faces a number of challenges (e.g., formalization, automation, scalability). For instance, the use of formal verification (formal methods community) is hindered by the state-space explosion problem, whereas schedulability analysis (real-time systems) is not suitable for behavioral properties. Moreover, current real-time implementations of robotic software are limited in terms of *predictability* and *efficiency*, leading to, e.g., unnecessary latencies. This is flagrant, in particular, at the level of locking protocols in robotic software. Such situation may benefit from major theoretical and practical findings of the real-time systems community. In this paper, we propose an interdisciplinary approach that, by joining forces of the different communities, provides a scalable and unified means to efficiently implement and rigorously verify real-time robots. First, we propose a scalable two-step verification solution that combines formal methods and schedulability analysis to verify both behavioral and real-time properties. Second, we devise a new multi-resource locking mechanism that is efficient, predictable, and suitable for real-time robots and show how it improves the latter’s real-time behavior. In both cases, we show, using a real drone example, how our approach compares favorably to that in the literature. This paper is a major extension of the RTCSA 2020 publication “A Two-Step Hybrid Approach for Verifying Real-Time Robotic Systems.”

## 1 Introduction

### 1.1 Context and Addressed Problem

Robotic systems are being increasingly deployed in human environments (e.g., home assistants, robotic surgery, autonomous-driving cars) and costly missions (e.g., space exploration). Most modern robotic systems are thus *safety critical*, due to the severe consequences of their possible failure, ranging from considerable economic losses to human injuries. This notion of “safety critical” is oftentimes tied to the *real-time* capabilities of the robot. For instance, *obstacle avoidance*, a classical ingredient of autonomous navigation, must be realized in real time, i.e., the robot must stop or navigate away *soon enough* not to collide with, e.g., a human on the field. In this paper, we focus on autonomous robots in this scope, which we simply refer to as real-time robots.

A real-time robot results from a tight coupling between software and hardware. The software, inherently complex, is mostly *component-based* [e.g., MAUVE (Gobillot et al., 2019), OROCOS (Soetens and Bruyninckx, 2005), and GenoM3 (Mallet et al., 2010)]. The software components, commonly known as *functional components*, collaborate while interacting with the hardware, typically a *multi-core embedded computer*, which we abbreviate as “MEC” hereafter, and a set of sensors and actuators. Each functional component implements complex algorithms, often organized in tasks, to perform some computations using the resources provided by the MEC. Computations results are communicated between components to close the perception–action loop and fulfill the robot’s missions.

Due to their safety-critical nature, exemplified above, it is crucial to guarantee that real-time robots behave safely and correctly *w.r.t.* the real-time constraints of the robotic mission, considering both their software implementation and MEC’s capabilities. The latter are usually limited, featuring only a small number of cores on which a large number of software tasks are assigned. These hardware limitations are due to the *size, weight, and power* (SWaP) considerations. For instance, we can see this in autonomous drones used in advanced research, e.g., Kamel et al. (2015); Khedekar et al. (2019); Chermprayong et al. (2019) (two cores) and Walter et al. (2018); Jeong et al. (2021) (four cores) and industry, e.g., the Quanser *QDrone* ^1^ (four cores). The complexity and constraints of the software–hardware couple, as introduced above, render providing sufficient guarantees on the correctness and safety of real-time robots a particularly hard research problem as explained hereafter.

The first major issue pertains to the “verification” practices within the robotic community. Roboticists usually rely on *scenario-based testing*, carried out in the field, or, to avoid field testing costs, by means of robotic simulators such as Gazeebo (Koenig and Howard, 2004) and MORSE (Echeverria et al., 2012). Unfortunately, scenario-based testing is inherently unreliable, as faulty scenarios may remain uncovered even by the heaviest and longest testing campaigns. Many examples in the literature corroborate the previous statement. For instance, Pecheur (2000) gave the details of a full-year test failing to detect a bug in a NASA experiment. Another example is reported by Kress-Gazit et al. (2011), where a software bug, while never occurring during thousands of hours of simulations and over 450 km of field tests, disqualified the autonomous vehicle *Alice* from the 2007 Defense Advanced Research Projects Agency (DARPA) urban challenge. More details on these two examples, as well as further examples, may be found in Foughali (2018), Chapter 1.

Besides, even if one assumes some sound verification approaches may efficiently replace scenario-based testing in robotics, a second major issue arises: mainstream robotic frameworks have little focus on real-time capabilities in the rigorous sense of the expression, making them unsuitable for real-time applications. The conclusions of Maruyama et al. (2016) provide a prominent example of this unsuitability *w.r.t.* the Robotic Operating System (ROS) (Quigley et al., 2009), the most popular robotic framework today. Recent attempts are made to switch to ROS2 which is under development^2^ with real-time issues still being investigated (Blass et al., 2021; Choi et al., 2021).

Two questions then immediately follow: What should scenario-based testing be complemented with in order to provide rigorous guarantees on the safety of real-time robots? And what should be done to provide acceptable real-time capabilities within robotic frameworks? The answer to either question requires multidisciplinary approaches at the crossroads of the robotic, formal methods, and real-time systems communities. We first give, for each question, a proposition that takes into account the interdisciplinarity of the question and discuss the related problems (Section 1.2). Then, we explain, through our contributions, how we concretize such propositions (Section 1.3).

### 1.2 Propositions and Difficulties

Proposition 1 Using rigorous verification techniques in robotics.

Scenario-based testing should be accompanied by mathematically sound approaches where important *behavioral properties* (e.g., liveness and safety) and *real-time properties* (e.g., schedulability and bounded response) are rigorously verified against a *model* faithfully representing the software–hardware couple that is the real-time robot. *Formal verification* and *schedulability analysis* belong to such approaches. Formal verification can deal with both behavioral and real-time properties, but its use in robotics is impeded by scalability issues. Indeed, if the formal technique is exhaustive (e.g., model checking), the *state-space explosion* problem is observed in real-world robotic systems, i.e., their state spaces are intractable because of their sheer complexity. On the contrary, if the formal technique is non-exhaustive, such as statistical model checking (SMC) (Legay et al., 2010), the properties can no longer be evaluated with certainty, but with some probability, which is not sufficient in critical missions [e.g., if a task in a component is hard real-time (HRT), its schedulability must be verified with certainty]. Finally, the literature on formal verification of robotics ignores MEC and operating system (OS) constraints, which restricts the results’ validity (Section 10). Likewise, the applicability of schedulability analysis to robotic systems is limited. First, its theoretical results are hardly generalizable to robotic tasks because the latter models are much more complex than the task models used in the real-time systems’ literature (Section 2). Second, schedulability analysis leaves other important properties such as behavioral properties unattended. The core of this proposition is to develop an approach that combines the advantages of both formal verification and schedulability analysis for a rigorous verification of real-time robots.

Proposition 2 Adapting real-time algorithms to robotic frameworks.

Typically, tasks in a real-time robotic application are *dependent* on each other, where the dependency stems from their need to perform computations and exchange data, and thus access the MEC’s resources concurrently. The way the exclusive access to resources is handled, i.e., *the real-time locking protocol* (Brandenburg, 2020) (the algorithm used to *lock* and *unlock* the MEC’s resources^3^ when accessed concurrently by real-time tasks), has a direct effect on schedulability and therefore real-time performance (more in Section 2 through Section 10). Mainstream robotic frameworks lack *predictable* (bounded blocking) and *efficient* (low-overhead) locking protocols (Section 2, Section 10). In other words, there is an urgent need to use a real-time locking algorithm that is (1) efficient, (2) predictable, and (3) suitable for robotics. Such suitability refers to, *inter alia*, managing resources in a *fine-grained*, *multi-resource*, *read/write* fashion with possible *mixed read–write requests* (Section 6.2). State-of-the-art fine-grained multi-resource protocols (from outside the robotic community) are promising candidates, yet none satisfies all the above three requirements. In particular, DGL, the multi-resource version of the real-time nesting locking protocol (RNLP) family (Ward and Anderson, 2012; Ward and Anderson, 2013; Ward and Anderson, 2014; Ward, 2016), suffers from efficiency drawbacks and does not support mixed read–write requests, whereas MRLock (Zhang et al., 2013) shows degraded predictability in corner cases (Section 6.3). The core of this proposition is to benefit from the advantages of DGL and MRLock in order to propose a new implementation of a predictable-and-efficient locking protocol that is suitable for real-time robots.

### 1.3 Contributions

From the analysis and observations made in Section 1.2 above, we establish a dependency between Propositions 1 and 2. Indeed, a predictable, efficient, and suitable locking protocol (Proposition 2) has, due to its direct effect on real-time performance, a direct consequence on Proposition 1 (e.g., a protocol with lower overheads and lower blocking bounds may lead to better schedulability, more in Section 8 and Section 9). Therefore, we depict our first contribution as an overall verification approach that remedies the problems discussed under Proposition 1. Then, we explain our second contribution as a solution to the problems discussed under Proposition 2 and show how we integrate such a solution in the overall verification approach in order to obtain better verification results (essentially better schedulability and tighter blocking bounds).

Our first contribution is the two-step verification approach presented by Foughali (2020), of which the current paper is an extension. We combine formal methods and schedulability analysis, where neither of the two is sufficient alone (Section 1.1). Our approach enables verifying both real-time and behavioral properties while taking into account the actual specificities of the robotic platform (mainly the MEC’s number of cores and scheduling policy). Furthermore, we provide a high level of automation, which makes our approach suitable for robotic programmers with no particular knowledge of formal methods or schedulability analysis. Step one focuses on guaranteeing schedulability with certainty. We develop a schedulability test for HRT robotic tasks, which belong to a (mixed-)critical application, under a fixed-priority (FP) preemptive policy and where resource sharing is handled using the global real-time locking protocol MSRP (Gai et al., 2001). If the original application, or a modified version achievable by, e.g., modifying tasks’ deadlines, together with the MEC’s number of cores satisfies this test, then schedulability of HRT tasks is guaranteed. This will be the basis of step two, where we verify, up to a high probability, other important properties less crucial than schedulability of HRT tasks. Such verification is done with SMC on formal models that we automatically generate from the robotic application, the number of cores, and the FP scheduler (altogether proven to satisfy schedulability for HRT tasks in step one). The approach is applied to a real autonomous drone system, developed using the robotic framework GenoM3, and the verification in step two is carried out using the formal framework UPPAAL-SMC (David et al., 2015).

Our second contribution boils down to LLAB, a *lock-less array-based* implementation of DGL, and R/W LLAB, its *task-fair multi-resource reader–writer* variant, as new *asymptotically optimal* and efficient real-time locking implementations that are suitable for robotics. We conduct a set of experiments on different platforms to show how the LLAB (resp., R/W LLAB) implementations have lower overheads than both DGL and MRLock while guaranteeing the same (resp., providing better) blocking bounds than DGL. Finally, we reiterate the two-step verification approach on the same drone system where we replace global MSRP with R/W LLAB and show how the new verification results confirm a better schedulability and tighter blocking bounds in the verified real-time robot.

### 1.4 Outline

The rest of this paper is organized as follows. In Section 2, we provide background on real-time robots and exemplify through presenting GenoM3 and an autonomous drone case study. Then, we present our first contribution in Section 3 through Section 5. In Section 3, we give examples of crucial properties in robotics and analyze the problems preventing their verification with formal methods or schedulability analysis independently. In Section 4, we detail our verification approach, where resource sharing is handled using global MSRP. Section 5 shows and discusses the results of applying our verification approach to the drone case study. Afterward, we present our second contribution in Section 6 through Section 9. In Section 6, we rely on the background given in Section 2 to show the limitations of the current locking choices in robotic frameworks and derive accordingly a set of requirements *w.r.t.* to the real-time locking protocol needed in robotics. Then, we show why new implementations of algorithms like DGL may fulfill such requirements while performing better than global MSRP. In Section 7, we present our LLAB implementation and its reader–writer variant R/W LLAB. Section 8 experimentally evaluates LLAB and R/W LLAB and compares their performance to that of other real-time locking protocols including DGL. We reiterate afterward the verification process on the same drone case study on new models integrating R/W LLAB and formally show the gains in schedulability and blocking bounds (Section 9). Finally, we compare our work to the state-of-the-art in Section 10 and conclude with possible directions of future work (Section 11).

This paper is an extension of the RTCSA 2020 publication “A Two-Step Hybrid Approach for Verifying Real-Time Robotic Systems” (Foughali, 2020). In particular, the second contribution and its integration in the verification process (Section 6 through Section 9) are new material.

## 2 Background

Robotic software is typically developed using dedicated component-based frameworks (Kortenkamp and Simmons, 2008). Each framework is coupled with a *middleware* (Elkady and Sobh, 2012), in charge of low-level primitives of, e.g., communicating with the OS. Though ROS (Quigley et al., 2009), the most popular robotic framework today (using its own middleware, called ROS-Com), is unsuitable for real-time robots^4^, a number of frameworks provide “real-time support” such as OROCOS (Bruyninckx, 2001), MAUVE (Gobillot et al., 2019), and GenoM3 (Mallet et al., 2010). Such support is provided through middleware where, contrary to ROS-Com, some real-time aspects are considered and analyzed: the OROCOS-RTT middleware (Soetens and Bruyninckx, 2005) for both MAUVE and OROCOS and the PocoLibs^5^ middleware for GenoM3^6^. In the remainder of this paper, we omit the term “middleware” to alleviate writing and reading alike and refer to the couple framework/middleware simply using the name of the framework and the term “framework,” which will thus include both the framework and its proper middleware. For instance, OROCOS will refer to the OROCOS framework using the OROCOS-RTT middleware, whereas GenoM3 refers to the GenoM3 framework using the PocoLibs middleware.

In this paper, all our models, analyses, and results are carried out on GenoM3 specifications. This is due to the main advantage of GenoM3 having automatic translations toward formal verification frameworks [e.g., to Fiacre/TINA (Foughali, 2017) and UPPAAL-SMC (Foughali et al., 2019b)] the soundness of which is mathematically proven (Foughali et al., 2019b), and that GenoM3 was the basis of our work in Foughali (2020) of which the present article is an extension. We will still point out the similarities between GenoM3 and the other real-time–oriented robotic frameworks throughout this section and discuss more their common limitations and how our contributions may apply to any of them in Section 10.

### 2.1 Robotic Software Specificities

We briefly present robotic software specificities using GenoM3 and a quadcopter case study.

A robotic software, which we call a *system*, is made of communicating components (Section 1.1). To account for timing constraints, a component encapsulates *periodic tasks*, in charge of its complex algorithms. The latter are organized within *services*. Because services are heavy and share resources, they are broken into small *pieces of code*, each attached to a state in a finite-state machine (FSM), hence the popularity of FSMs in robotics. Thus, there are four “levels” in a system (from the lowest to the highest): pieces of code, services (FSMs), tasks, and components.

Though not unanimous in robotics, the above organization is used by most real-time–oriented robotic frameworks with subtle differences (e.g., while MAUVE and OROCOS confound components with tasks, i.e., a component is a task, GenoM3 preserves both levels). Note that since there is no standard terminology for most levels, the one we use is that of GenoM3.

We provide a generic informal description of GenoM3 with a focus on concurrency and real-time aspects. A more formal example using timed automata is given in Section 4.1. Note that this description is simplified for readability and to remain in the scope of this paper (e.g., control tasks and aperiodic tasks are excluded).

**FIGURE 1:**
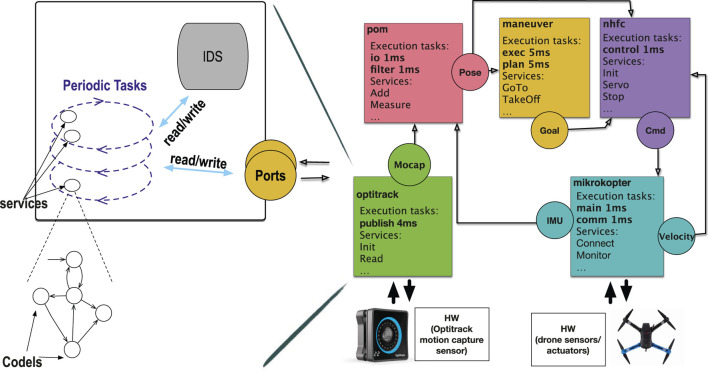
A drone example showing some services of the quadcopter case study (right) and a generic GenoM3 component (left).

The organization of a component is shown in Figure 1 (left), where we can see the three component “levels” described above. Pieces of code are called *codels*. Each codel, attached to a state of a service FSM, has a worst case execution time (WCET). By abuse of terminology, FSM states are simply called codels. Each task *t*, featuring a period, is in charge of a set of services *S*_*t*_. We say that each service *s* ∈ *S*_*t*_ is **a** service of *t*, and *t* is **the** task of *s* (*s* cannot belong to any *S*_*t*′_ with *t*′ ≠ *t*). To perform their computations, codels share the internal data structure (IDS) of the component. Finally, *ports* are used to communicate with other components and are thus accessible by the codels in all components that use them.

Codels are thus *critical sections* that usually have short execution times (see the drone example in Section 2.2). The execution of a codel is subject to a prior locking of a *set of resources* (multiple resources), each resource being an IDS fragment or a port. A resource may be locked in shared (read-only) or exclusive (write) mode.

#### 2.1.1 Behavior

We briefly explain how a component evolves in a top-down fashion (from tasks to codels), following the *scheduler-agnostic* semantics developed by Foughali et al. (2019b).

The component is piloted by an external *client* that *requests* services. Each task *t*, at each period, executes only the services previously requested by the client (among services in *S*_*t*_) sequentially. When a service finishes executing, the task informs the client by sending a *report*. Communication between clients and tasks is abstracted in the rest of this paper for readability and simplicity.

Each service FSM has at least two codels: start (at which the first execution begins) and ether (the termination point). A service execution ends when either (i) codel ether is reached (service is terminated) or (ii) another codel c is reached after taking a *pause transition*, i.e., a transition labeled *pause* [see the abstract FSM in Figure 1 (left)]; we say then the service is *paused* and refer to c as a *pause codel*. In the latter case, the service is resumed, at the next period of its task, starting from c.

#### 2.1.2 Concurrency

Tasks (in a system), each of which executes its requested services sequentially (see the previous paragraph), are run as parallel threads (assuming enough cores are available). To maximize parallelism, access to shared resources is handled at the codel level: resources (ports or fields of the IDS) that a codel needs for its execution are statically defined, so two codels *in conflict* (using at least the same port or the same IDS fragment) may not execute in parallel (simultaneous readings are allowed). Thus, while executing its requested services, a task needs to busy-wait (*spin*) when one of such services reaches a codel in conflict with another codel, in another service being executed by another task concurrently. Following this low-level concurrency model, a codel may be either thread safe (TS) (not in conflict with any codel) or thread unsafe (TU) (otherwise). Because of ports sharing, codels in conflict may belong to different components (example in Section 4.1).

#### 2.1.3 Specification and Templates

While we content with graphical illustrations of GenoM3 systems, the latter are actually specified textually. Each component is written in a *dotgen* (*.gen*) file, in which tasks, services, and codels are specified. A system may be then built by #-including the dotgen files of the different components in another dotgen file.

*Templates* transform dotgen specifications into Tool Command Language (Tcl) structures for automatic generation purposes. The robotic programmer can access all information in the dotgen file (e.g., task periods, FSM, and codel WCET), manipulate it, and generate a text file in any format accordingly. We have used this mechanism in previous work to automatically generate formal models (Foughali, 2018). In Section 4, we give examples of templates developed to automatize the two-step approach presented in this paper.

### 2.2 Case Study

To validate our approach, we use the quadcopter case study from LAAS-CNRS. Figure 1 (right) shows its GenoM3 organization in which some names are modified for simplicity. The system contains five components collaborating to achieve autonomous aerial navigation. We give a high-level description (in terms of components and ports) on how these components collaborate [the interested reader may refer to Foughali (2017) for more details on each component].

Component mikrokopter processes data from the inertial measurement unit (IMU) and the propellers’ sensors and uses them to write the current IMU and velocity to ports **IMU** and **Velocity**, respectively. Component optitrack processes data from the Optitrack motion capture system and writes them to port **Mocap**. Component pom reads the IMU and captures position from, respectively, ports **IMU** (mikrokopter) and **Mocap** (optitrack), to which it applies an unscented Kalman filter (UKF) to compute the estimated position of the drone that it writes to port **Pose**. Such position is fed to (i) maneuver, which uses it to compute an intermediary goal position that it writes to port **Goal**, and (ii) nhfc, which uses it, together with the current **Velocity** (from mikrokopter), to compute and update, in port **Cmd**, the velocity to reach the intermediary goal position (from **Goal** in maneuver). Finally, the perception–action loop closes as mikrokopter reads the updated velocity in **Cmd** (nhfc) and applies it to the drone propellers.

In the quadcopter case study, tasks run at high frequencies (most at 1 kHz), and critical sections, typically short (less than 50 *μ*s), share more than 30 resources (IDS fragments and ports). Hardware-wise, the drone is controlled by an ODROID-XU3 MEC, featuring an ARM-based quad-core CPU. This low number of cores is dictated by the SWaP considerations as explained in Section 1.1.

## 3 The Verification Challenge

In this section, we explain the importance of rigorous verification of real-time robots and detail their challenges using the drone example presented in Section 2.2.

If the drone software fails, the drone may crash, inducing economic costs and/or human injuries. We give examples of crucial properties that must be verified to avoid such failure and explain why their verification is particularly challenging.

### 3.1 Properties of Interest

The drone system has three critical components: mikrokopter, nhfc, and pom. That is, tasks in these components are HRT: each must always finish executing within its period; otherwise, the drone may crash. It follows that the schedulability property must be proven always true for these tasks, for all possible scenarios. In the remaining components (less critical), tasks are allowed to miss their deadlines. However, it is still important to verify that they are, e.g., exempt of starvation, that is, being, at some point, delayed forever by critical tasks monopolizing resources. For example, in tasks in maneuver, such starvation would make the drone hover forever without fulfilling its mission (as it may not navigate to a final goal position). These tasks must thus not starve, but also, ideally, respect their deadlines for a timely fulfillment of the mission.

### 3.2 Difficulties

Now, in order to verify these properties, using model checking (or SMC) or schedulability analysis independently proved insufficient in robotics in general and on this drone system in particular.

#### 3.2.1 With Model Checking/SMC

Model checking does generally not scale with complex robotic applications. For instance, we show in Foughali et al. (2019b) that although it performs well on the *stationary flight application* (i.e., component maneuver is excluded), model checking with state-of-the-art tools fails to scale on the *navigation application* involving all the five components (Figure 1), with eight tasks and over 20 services broken into more than 80 codels. In the same work (Foughali et al., 2019b), we use SMC to verify properties up to a high probability. Though SMC provides better guarantees than scenario-based testing, it is not suitable for the schedulability property of HRT tasks which must be proven with certainty.

Another problem of model checking (and generally formal verification) in robotics is that extending formal models with scheduling algorithms usually penalizes their scalability because of (i) preemption and/or (ii) the necessity to create large models to handle schedulers (Foughali and Hladik, 2020). For the drone navigation application, the integration of schedulers in formal models (which already do not scale as explained above) produces new formal models that still do not scale, even when preemption is not allowed.

#### 3.2.2 With Schedulability Analysis

From a real-time analysis point of view, we focus on three levels in GenoM3 (and generally robotic) systems: the tasks level, the services level, and the codel level (components are abstracted as tasks map to cores). Robotic task models are thus more complex than those usually considered in real-time analysis: a robotic task executes, at each period, a sequence of services each comprising a sequence of codels with possible spinning and/or preemption between them, rather than one job whose WCET is known. A particular problem is the computation of the WCET of tasks, which is practically intractable. Indeed, besides the fact that a TU codel (Section 2.1.2) may remain infinitely blocked waiting for resources (robotic frameworks do not guarantee the absence of starvation), the sequence of codels to execute in services by a task may differ from a period to another depending on, e.g., which services are requested (Section 2.1.1). Another problem is that even if such sequences’ WCETs are somehow obtained, theoretical results of schedulability analysis in the literature are still unusable because the preemption model in robotics is also different (more in Section 4.1). Finally, schedulability analysis provides no guarantees on other properties excluding schedulability.

## 4 A Two-Step Hybrid Approach

Our approach combines both formal verification, by means of SMC, and schedulability analysis to achieve scalable rigorous verification of crucial properties in robotics. We divide properties into two types: *Type I* covers properties that must be verified with certainty (schedulability of all HRT tasks), while *Type II* comprises properties that may be verified with a high probability (e.g., absence of starvation in less critical tasks). On that basis, the key idea is the following. Since model checking does not scale, we may use SMC for *Type II* properties, but only once properties of *Type I* are verified with certainty. Thus, we first check whether we can guarantee properties of *Type I* using schedulability analysis. This is the first step of our approach, which takes into account the actual number of cores on the MEC and a scheduling policy (Section 4.1). If step one is conclusive, an UPPAAL-SMC model of the considered application, number of cores, and scheduler (already proven to satisfy properties of *Type I* in step one) is generated. On such formal model, we verify by means of SMC properties of *Type II*, which concludes the second step of our approach (Section 4.2).

### 4.1 Step One: Schedulability Analysis

Our approach is based on response time analysis (RTA). First, we compute the tasks’ WCETs, taking into account delays caused by mutual exclusion over resources (Section 4.1.1). Then, we compute the tasks’ worst case response time (WCRT) considering the concurrency over cores (Section 4.1.2).

#### 4.1.1 Computing Tasks’ WCET

**FIGURE 2:**
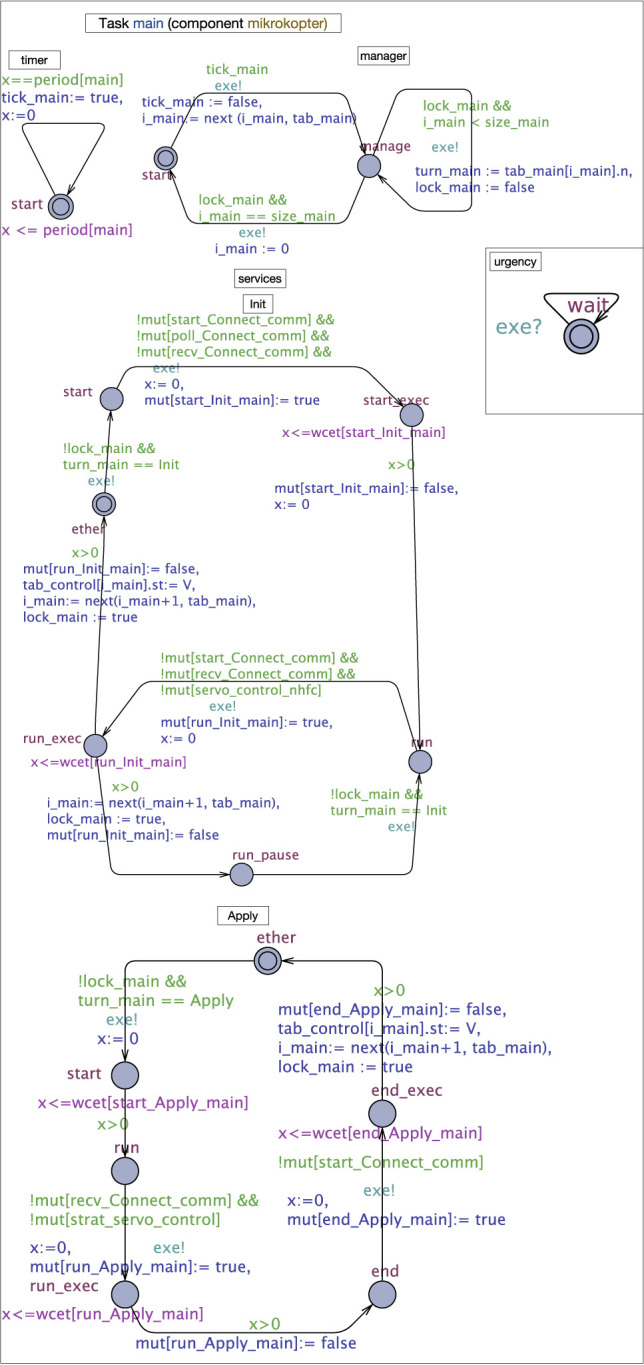
Simplified UPPAAL model of task main in component mikrokopter. Process *Urgency* does not belong to any component and is added to enforce urgencies, i.e., prevent unnecessary lazy waits (the receiver edge “exe?” is always ready).

In the following, we explain more where the difficulty of computing task WCET (Section 3.2) comes from using the UPPAAL formal model of the GenoM3 task main (component mikrokopter) of the drone (Figure 1) shown in Figure 2. This model, automatically generated, is proven correct *w.r.t.* GenoM3 semantics (Foughali et al., 2019a; Foughali et al., 2019b). The model is simplified for readability purposes.

Each timed automaton (TA) in UPPAAL, made of *locations* and *edges* connecting them, and possibly having a *clock* *x*, is called a *process*. Time *invariants* (in purple) may be associated with locations, and edges may have *guards* (in green) and *operations* (in blue). Processes are arranged to fit with the “layers” view given in Section 2.1: the task layer, composed of processes *timer* and *manager*, the service layer, where each underlying GenoM3 service FSM is mapped to an UPPAAL process, and the codel layer, where codels are locations in service processes. Figure 2 shows that task main has two services: *Init* and *Apply*.

Shared variables and functions are used by processes to communicate. The array *tab*_*t* holds the names and “statuses” of all services of task *t*. Each of its cells contains two fields: *n*, a service name, and *st*, the service status that may be either *R* (requested by a client) or *V* (for “Void,” otherwise). The timer of *t* gives at exactly each period a signal, through variable *tick*_*t*, to the manager to start execution, by taking the edge *start* → *manage*. The operation of such edge searches, through function *next*(), for the index of the next requested service in *tab*_*t* (having status *R*) starting at index *i*_*t* (initially 0) and stores the result in *i*_*t* (the size of *tab*_*t* if such service does not exist). At location *manage*, the manager executes the requested services sequentially: variables *lock*_*t* and *turn*_*t* are used to pass the control to the next service to execute (computed previously through function *next*()). When such service finishes execution, by either terminating^7^ (e.g., edge *end*_*exec → ether* in service *Apply*) or pausing (e.g., edge *run*_exec → run_*pause* in service *Init*), it computes the index of the next service to execute and gives the control back to the manager. And so, the control passes back and forth between the manager and the requested services until each of the latter has executed once (detected when *next*() hits the bottom of *tab*_*t*), so the manager transits back to *start* and awaits the next period.

Now, at the codel level, a codel c in a service *s* is represented by either one location c (if it is TS) or two locations *c* and *c_exec* (otherwise) plus a location *c_pause* if such codel is targeted by a pause transition in the underlying GenoM3 specification. The WCET of c is represented with an invariant *x ≤ wcet[c_s_t]* on location *c* ( *c_exec* if c is TU), where *wcet* is an array of all codels’ WCETs indexed with unique identifiers. The array of Booleans *mut* is used to handle concurrency: it tracks the execution of TU codels in the system. Therefore, guards on edges *c → c_exec* ensure *c* does not start executing unless no codel in conflict with *c* is currently executing, witnessed by the falseness of the corresponding fields in *mut* . For instance, codel run of service *Apply* is in conflict with codel recv (in service *Connect*, executed by the other task comm in mikrokopter), and codel start (in service *servo* of task control in component nhfc), which explains the guard on the edge *run → run_exec* in process *Apply*. If such guard is true, codel run starts executing by taking *run → run_exec* through which it turns its own field in *mut* to true to prevent, in turn, codels in conflict with it to execute.

This example shows the complexity of GenoM3 (and generally robotic) tasks. From a real-time analysis perspective, we identify two problems. First, the WCET of a sequence of codels (which a task executes) is possibly infinite because we do not know beforehand how long a TU codel needs to wait to secure the resources it needs (the blocking time). Second, even if we bound such blocking time, it is practically infeasible to compute by hand the WCETs of all possible sequences: for instance, summing the WCETs of all codels in all services in a task (assuming we bound and include blocking bounds in TU codels’ WCETs) would be a naive solution (such sum would be a coarse overapproximation that will likely prevent finding a feasible schedule). We propose a solution for both problems by, respectively, (i) an implementation to bound blocking times for TU codels and (ii) an algorithm to compute the WCET of a task by traversing all possible codel sequences. We explain how the solution can be automated.

##### 4.1.1.1 Bounding TU Codels’ WCET

We propose an implementation to enable computing a blocking bound *B*_*c*_ (on the time needed to acquire resources, *i.e.*, IDS or ports) of any TU codel c. Then, we get the actual WCET of c by summing its WCET (from the GenoM3 specification) with *B*_*c*_.

Listing 1:
Generating the largest WCET of TU codels per task.

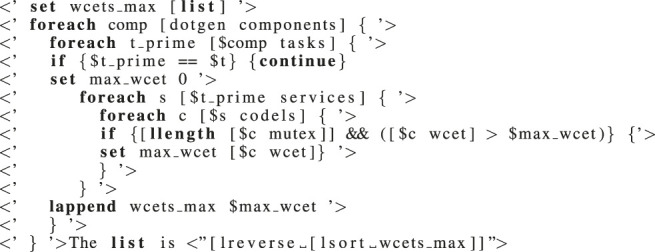

The implementation is inspired from multiprocessor resource-sharing protocols. Brandenburg (2011) reviewed a number of such protocols, mainly categorized into *spin-based* (*busy-waiting*) and *suspension-based*, and pointed out that the former are easier to implement and perform better than the latter when durations of critical sections are short. As we explained in Section 2, FSMs in robotics are designed to reduce the times of locking shared resources, which makes spin-based protocols suitable to our case. Actually, the previous reasoning fits with the reality of spinlocks being widely used in robotics (e.g., ROS and GenoM3 systems). At first, we use the global MSRP protocol (Gai et al., 2003). In a nutshell, a TU codel c appends itself to an FIFO queue, and its thread is spinlocked until c gets access to shared resources, and spinlocked threads are non-preemptible. TS codels are not concerned as they are in conflict with no other codel in the system (Section 2.1.2). The direct disadvantage of this approach is that all TU codels compete for the shared resources as a whole, which reduces the overall parallelism of the system (it is possible for a TU codel c to be blocked by another TU codel c’ in the FIFO queue even though c and c’ are not in mutual conflict). In Section 9, we will use our new fine-grained algorithm R/W LLAB (which we devise and evaluate in Section 6 through Section 8) which efficiently and predictably overcomes this disadvantage.

Let us compute *B*_*c*_ of a TU codel c in a service *s* in a task *t*. We assume there are *n* tasks and *m* cores (*m*<*n*). In worst case scenarios, the thread trying to execute c spins after already *m* − 1 threads are in the spinlock FIFO queue (for accessing shared resources). Since each thread corresponds to a GenoM3 task that (i) is sequential and (ii) spins only when trying to execute a TU codel, the first *m* − 1 entries of the FIFO are occupied by TU codels each in a distinct GenoM3 task, different from *t*. In the worst case, each *t*′ of the *m* − 1 tasks already spinlocked is trying to execute TU codel c’ with the largest WCET among the TU codels of all services in *t*′. Thus, *B*_*c*_ is upper-bounded by the sum of the WCET of codels c’. To get that sum, we proceed as follows: (1) For each task *t*′ ≠ *t*, we find, within all its services, the largest WCET of all TU codels. (2) We sort, in decreasing order, the values found in (1). (3) *B*_*c*_ is equal to the sum of the first *m* − 1 values sorted in (2).

Once *B*_*c*_ is computed, we sum it with *WCET*_*c*_ (the WCET of codel c given in the GenoM3 specification) to get the *actual* WCET of c (including the blocking bound). To make codels’ actual WCET computations accessible to robotic programmers, we make use of the template mechanism (Section 2.1.3). We give in Listing 1 an example that performs steps (1) and (2) of the algorithm above and then writes (to a file) the list output by (2) for any TU codel in any service in task *t*. The template generator evaluates everything enclosed in < ‘ ’ > (resp., < “ ” > ) in Tcl without output (resp., and outputs the result) and outputs the rest as is. Line 4 excludes task *t*, and line 8 conditions considering codel c only when it is TU through the non-emptiness of the field [*$c mutex*], a ready-to-use list containing all codels in conflict with c. The last line writes to a file the list after sorting it in decreasing order.

Thus, at the end of these computations, we have the actual WCET of all codels, which we call simply WCET in the remainder of this section and throughout the following section with the verification results (that is, the WCET provided by GenoM3 if c is TS or summed with *B*_*c*_ if c is TU). Our approach to compute *B*_*c*_ is generic and may thus be pessimistic in some cases. For instance, if the scheduler is partitioned, some of the *m* − 1 largest elements of *wcets*_ max (Listing 1) may belong to tasks allocated to the same core as *t*, and thus, *B*_*c*_ is overestimated. However, this genericity brings a valuable advantage. Indeed, since the computation is affinity-independent, the roboticist performs this step only once and, if some HRT tasks do not pass the schedulability test (Section 4.1.2), may try to find a better affinity by reallocating tasks based on the timing constraints already computed (the affinity does not affect such constraints). This is explained further in Section 5.

##### 4.1.1.2 Deducing Tasks’ WCET

We call each possible (full) codel sequence executed by task *t* a *hyperjob*. The largest WCET of all hyperjobs in *t* is then simply the WCET of *t*.

Therefore, to compute the WCET of *t*, we proceed as follows: (1) For each service *s* in task *t*, we sum the WCETs of codels involved in each possible path (starting at either codel start or some pause codel and ending at either ether or some pause codel). (2) We find, for each *s*, the value of the largest among the sums computed in (1). (3) We sum the values found in (2). (4) We repeat (1), (2), (3) for all tasks in the GenoM3 system. Thus, this algorithm will give the maximum time to execute the longest possible path in all services in *t*, which corresponds to the largest WCET of all possible hyperjobs in *t* (i.e., the WCET of *t*).

The above algorithm being classical in model checking, the idea is to benefit from the already existing UPPAAL template (Foughali et al., 2019b) to achieve it. Yet, we know that the overall UPPAAL model of this application does not scale. The good news is, however, we do not need to consider the system as a whole: since WCETs are now known for all codels, we may adapt service processes of task *t* to allow computing the maximum time of their possible paths [step (1) above] without considering the rest of the system.

First, locations *c*_*exec* are no longer needed: location *c* is enough, the invariant bound of which is the WCET of codel c (Section 4.1.1.1). That being done, interactions of each service with services outside *t* cease to exist (since bounds *B*_*c*_ are now included in TU codels’ WCET, all guards and operations involving the *mut* array are removed). Then, we (i) make all *ether* and *c*_*pause* locations *urgent* (time cannot progress at them) and add, to each service process of *t*, a clock *y* reset to 0 at all edges leaving *ether* or *c*_*pause* locations. This way, *y* tracks the time of each possible path from location *start* (or any *c*_*pause* location) to location *ether* (or any *c*_*pause* location). We have thus what we need for step (1) of the algorithm above and may remove all the remaining non-clock guards and operations in the services of *t*. It follows that there are no more interactions between any service process in *t* and the rest of the system, which means we can obtain the WCET for each possible path in each service separately.

**FIGURE 3:**
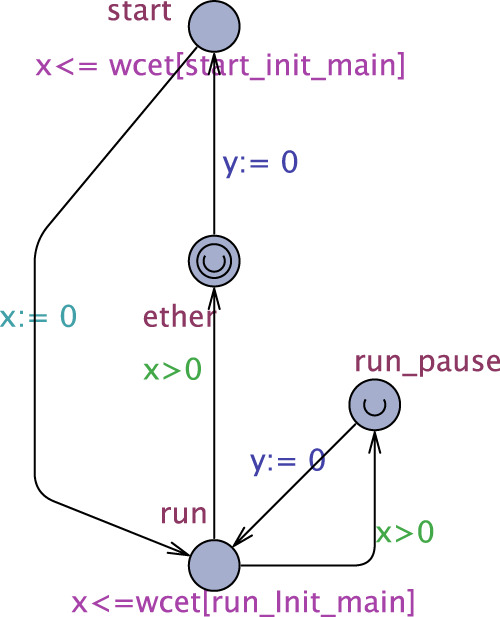
Modified process of *Apply* for WCET task computation.

Figure 3 shows the result of these changes to the UPPAAL process of service *Apply* (Figure 2). Now, all we need to do is ask UPPAAL for the maximum value of clock *y* at location *ether* and each location *c*_*pause* using the UPPAAL query pattern sup{*p*.*l*} : *p*.*y* (with *p* being the process name and *l* the location name), store the results, and repeat the operation for each service in task *t*, which corresponds to step (1) of the algorithm above. Then, we perform (2) and (3) and then repeat the whole process for all other tasks [step (4)] to get the WCET of all tasks in the GenoM3 system.

#### 4.1.2 Analysis

Once the tasks’ WCETs are computed, we compute their WCRT for RTA. We recall that schedulability tests from the literature are not applicable to robotic tasks even when they take memory-sharing into account. For instance, standard task and scheduling models assume a task executes only one job at each release. This means that if we use available tests, we should treat each hyperjob in each task *t* as a regular job and, since such hyperjob is likely to include a TU codel, make it non-preemptible (Section 4.1.1.1). Consequently, we will most likely end up with a set of non-preemptible tasks, which renders preemptive scheduling useless.

**FIGURE 4:**
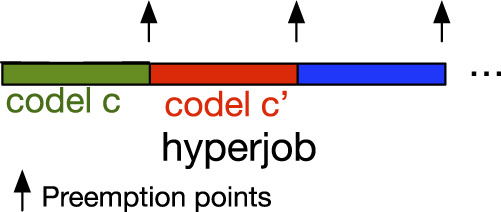
Hyperjob model.

Thus, we need to perform schedulability analysis based on the model in Figure 4: each hyperjob may be preempted at the end of each codel. The reason for this is rather straightforward: in robotics, elementary pieces of code (codels in GenoM3) are designed by roboticists as the smallest pieces (of the algorithm they belong to) that must be performed with no intermediary perturbations. TU codels present another feature that consolidates the rationale of codels’ non-interruptibility: their interruption may compromise their memory-dependent computations.

##### 4.1.2.1 Scheduling Assumptions

We use a partitioned FP scheduler. There are two main reasons. First, partitioned FP is very popular in domains related to robotics, such as automotive systems [e.g., in AUTOSAR (Wieder and Brandenburg, 2013)], since it removes the cost of task migration. Second, global schedulers are known to perform poorly compared to partitioned ones, even though this might result from over-pessimism of tests in global approaches (Gracioli et al., 2013).

For the sake of analysis, we introduce a first real-time model on which we will carry out schedulability analysis. The task set of the robotic system is the union of HRT and less critical tasks *τ* = *τ*_*h*_ ∪ *τ*_*l*_. There are two priorities: *pr*_*h*_ (resp., *pr*_*l*_), the high (resp., low) priority, assigned to all tasks in *τ*_*h*_ (resp., *τ*_*l*_). The platform features *m* cores *C*_1_… *C*_*m*_ (*m* < |*τ*|). Let *Aff*_*i*_ ⊆ *τ* be the affinity of core *C*_*i*_, that is, the set of tasks allocated to it. Then, *Aff*_*i*_ = *Aff*_*il*_ ∪ *Aff*_*ih*_ where *Aff*_*il*_ = *τ*_*l*_ ∩ *Aff*_*i*_ (resp., *Aff*_*ih*_ = *τ*_*h*_ ∩ *Aff*_*i*_) is the set of low (resp., high) priority tasks allocated to *C*_*i*_. Since the algorithm is partitioned, each task is statically allocated to only one core, that is, *∀i*, *j* ∈ 1..m, *i* ≠ *j*: *Aff*_*i*_ ∩ *Aff*_*j*_ = ∅. The size of the queue of *C*_*i*_ is equal to the cardinality of its affinity |*Aff*_*i*_|.

Following the model in Figure 4, a task is a set of hyperjobs *t* = {*hj*_1_…*hj*_|*t*|_}. A hyperjob is an ordered set of codels *hj* = {*c*_1_…*c*_|*hj*|_}. If a codel c belongs to a hyperjob in *t*, we may say simply that c belongs to *t*. When needed, we use the superscript (*t*) to denote that a hyperjob or a codel belongs to task *t* and the double subscript *jk* to denote that a codel *c*_*j*_ belongs to hyperjob *hj*_*k*_. Superscripts/subscripts are omitted when unnecessary, unimportant, or clear in the context.

This real-time model is deliberately specific to our approach, as it integrates the bounding of blocking times performed in this step. Further in this paper (Section 6), we will introduce a generic real-time and locking model for GenoM3 based on which we devise a new locking mechanism to replace MSRP.

##### 4.1.2.2 Computing Tasks’ WCRT

At each period *P*_*t*_, one (depending on the evolution of the system) of the hyperjobs in *t* is to be executed. The WCRT of *t* defines an upper bound on the time separating the moment *a*_*i*_, at which a hyperjob *hj*_*i*_ is activated (arrives in a core queue), and the moment *f*_*i*_, at which it finishes its execution and frees the core, that is, *WCRT*_*t*_ = *max*_*i*∈1‥|*t*|_(*f*_*i*_ − *a*_*i*_) (Eq. 1). Let *r*_*i*_ ∈ [*a*_*i*_, *f*_*i*_) be the moment *hj*_*i*_ is *released*, that is, a core is given to it and it starts to execute (*f*_*i*_ is excluded because *hj*_*i*_ cannot execute in zero time). By inserting *r*_*i*_ in Eq. 1, we get *WCRT*_*t*_ = *max*_*i*∈1‥|*t*|_(*f*_*i*_ − *r*_*i*_ + *r*_*i*_ − *a*_*i*_) which we may upper bound *WCRT*_*t*_ ≤ (*max*_*i*∈1‥|*t*|_(*f*_*i*_ − *r*_*i*_) + *max*_*i*∈1‥*n*_(*r*_*i*_ − *a*_*i*_)) (Ineq. 2). Now, we know that the left-hand operand of the right-hand side of Ineq. 2 is the WCET of *t* which we already computed in Section 4.1.1. We call the remaining operand the *worst case waiting time* *WWT*_*t*_ = *max*_*i*∈1‥|*t*|_(*r*_*i*_ − *a*_*i*_) (Eq. 3). Therefore, *WCRT*_*t*_ ≤ *WWT*_*t*_ + *WCET*_*t*_ (Ineq. 4).

A hyperjob *hj* of a high-priority task *t* allocated to core *C*_*i*_ (*t* ∈ *Aff*_*ih*_) worst position in the prioritized queue of *C*_*i*_ is equal to |*Aff*_*ih*_|. The worst waiting time of *hj* corresponds to this very position (hyperjobs of tasks in *Aff*_*ih*_, having the same priority *pr*_*h*_ as *t*, are already in the queue, so *hj* has to wait for them to finish). Now, in this worst situation, the worst case is when the hyperjob at the head of the queue cannot start immediately as a low-priority task hyperjob *hj*′ is still not preempted (we recall that preemption points are set at the end of each codel, Figure 4). It follows that the worst waiting time for *hj* is equal to the sum of the WCET of all |*Aff*_*ih*_| − 1 hyperjobs (each belonging to a task *t*′ ∈ *Aff*_*ih*_\{*t*}) in the queue plus the WCET of the codel of *hj*′ being currently executed. We maximize such worst waiting time for all hyperjobs in *t* to get *WWT*_*t*_ (see Eq. 3). To account for the waiting needed for high-priority hyperjobs, we maximize the WCETs of all hyperjobs in each task *t*′ ∈ *Aff*_*ih*_\{*t*} and sum them (1). Then, we add to the value obtained in (1) the waiting for preemption by maximizing the WCET of codels in low-priority tasks *t* ^″^ ∈ *Aff*_*il*_ (2). (1) is simply the sum of the WCET of tasks *t*′ ∈ *Aff*_*ih*_\{*t*}, and in (2), we add the WCET of the longest codel in tasks *t* ^″^ ∈ *Aff*_*il*_, which gives us the following bound for any task *t* allocated to core *C*_*i*_:

$$WWT_{t}\leq \Sigma_{t^{\prime }\in Aff_{ih}\backslash \left\{t\right\}}WCET_{t^{\prime }}+max_{\begin{array}{c} l\in 1‥|t^{\prime \prime }|\\ k\in 1‥|hj_{l}^{t^{\prime \prime }}| \end{array}}^{t^{\prime \prime }\in Aff_{il}}\left(WCET_{c_{kl}^{t^{\prime \prime }}}\right)\;\;\left(Ineq.\;\;5\right).$$

We sum *WWT*_*t*_ with *WCET*_*t*_ to upper bound *WCRT*_*t*_ (see Ineq. 4). Finally, we state the schedulability test for HRT tasks *∀t* ∈ *τ*_*h*_: *WCRT*_*t*_ ≤ *P*_*t*_ (Ineq. 6).

While pessimistic, this test is sufficient: if the maximum time a task *t* needs from its activation to its end is less than its deadline (period), then *t* is schedulable. We trade off optimism for *sustainability*: Burns and Baruah (2008) showed that RTA-based FP schedulability tests are sustainable in the sense that they remain valid even if some tasks manage to execute in less than their WCET.

### 4.2 Step Two: Formal Verification

If all HRT tasks in the GenoM3 system pass the schedulability test in step one, we may verify other—less critical—properties using SMC. We automatize the generation of UPPAAL-SMC models by extending the template presented by Foughali et al. (2019b).

First, we make sure that the WCET computations, made with the help of UPPAAL (Section 4.1), still hold in UPPAAL-SMC models. This is a simple proof. As shown by Foughali et al. (2019b), the only difference between UPPAAL-SMC and UPPAAL models is at the level of services, where non-deterministic edges may have custom probabilities. To give an example, let us get back to Figure 2. In process *Init*, there are two edges out of location *run*_*exec*. In UPPAAL, these edges are equiprobable (chances to take one or the other are equal). In UPPAAL-SMC, one may use custom probabilities (that sum to one) on such edges, a mechanism which we exploited in Foughali et al. (2019b) to insert experiment-based probabilities. Now, *w.r.t.* the computations made in Section 4.1, this difference has no impact since, for HRT tasks, we need to explore all paths anyway, no matter how big or small is the probability to take each of them. Second, we need to integrate the global MSRP protocol in the verified model and use the original WCETs of codels. Third, we need to integrate the FP scheduler in the UPPAAL-SMC model and automatize it in the template.

For readability, the technical details on how (i) the UPPAAL-SMC model is extended with the FP scheduler and (ii) the obtained formal model is automatized in a template are omitted in this paper, but the interested reader may refer to Foughali (2020) (Section III.B).

## 5 Results

We apply our approach to verify important properties on the drone navigation under a partitioned FP policy and the number of cores on the drone MEC (quad-core ODROID-XU3, Section 2.2).

### 5.1 Step One

We comply with the notation given in Section 4.1: *m* = *4* (number of cores), *τ*_*h*_ = {*main*, *comm*, *io*, *filter*, *control*} (the set of HRT tasks, i.e., high-priority tasks, those of the critical components mikrokopter, pom, and nhfc), and *τ*_*l*_ = {*publish*, *plan*, *exec*} (the set of low-priority tasks, those of components optitrack and maneuver). Following the steps given in Section 4.1.1, we compute the actual WCET of all TU codels in the system, update such WCET accordingly, and then compute the WCET of the five HRT tasks in the system (Table 1). For each of the remaining three tasks, we identify the codel having the largest WCET (Table 2)^8^. We recall task periods in Table 3.

**TABLE 1:** WCET of HRT tasks.

HRT task	WCET (ms)
main	0.51
comm	0.47
io	0.68
filter	0.55
control	0.52

**TABLE 2:** Longest-codel WCET in low-priority tasks.

Task	WCET of longest codel (ms)
publish	0.3
plan	0.4
exec	0.4

**TABLE 3:** Task periods.

Task	Period (ms)	Task	Period (ms)
main	1	control	1
comm	1	publish	4
io	1	plan	5
filter	1	exec	5

An issue that arises is how to allocate tasks to cores. It stems from the bin-packing problem, known to be NP-hard. In this paper, the way we allocate tasks is inspired by the *first-fit decreasing* heuristic. We start by allocating *m* high-priority tasks (in *τ*_*h*_) to the *m* cores and then repeat until all tasks in *τ*_*h*_ are allocated. Then, we do the same for low-priority tasks (thus allocation is by *decreasing* priority). The *first-fit* part is left to after running the schedulability test on HRT tasks (if such test fails). This allocation is not exactly what the original heuristic does, but in our case, it intuitively tends to reduce the WCRT of HRT tasks in the application. Indeed, such WCRT increases with the number of HRT tasks allocated to the core (Ineq. 5), and so allocating first HRT tasks minimizes the maximum number of HRT tasks allocated to a core *C*_*i*_, upper-bounded by ⌈*τ*_*h*_/*m*⌉. The (decreasing) affinity we start with is given in Table 4.

**TABLE 4:** Initial affinity.

Core	Affinity
*C*_1_	{*main*, *comm*}
*C*_2_	{*io*, *plan*}
*C*_3_	{*filter*, *publish*}
*C*_4_	{*control*, *exec*}

We are now ready for schedulability analysis: we apply Ineq. 5 (using the values in Tables 1, 2) for each task *t* to upper bound *WCRT*_*t*_ and then compare the latter with the period *P*_*t*_ in Table 3 (Ineq. 6). The results (Table 5) show that all HRT tasks pass the schedulability test except for task io, whose WCRT is 80 *μ*s larger than its period.

**TABLE 5:** WCRT of HRT tasks considering the initial affinity (Table 4).

HRT task	WCRT (ms)
main	0.98
comm	0.98
io	1.08
filter	0.85
control	0.92

At this point, we may try to change the affinity without modifying the decreasing pattern (no more than two HRT tasks per core). Here, the genericity of the approach in Section 4.1.1.1 allows us to reason only using the timing constraints in Tables 1, 2, which remain valid regardless of the chosen affinity. We notice that, by permuting the allocation of low-priority tasks publish and plan, all HRT tasks pass the schedulability test (Table 6). This new affinity guarantees schedulability for all HRT tasks in the system (Table 7) and will be thus the basis of step two.

**TABLE 6:** New affinity (by permuting tasks in blue in the initial affinity in Table 4).

Core	Affinity
*C*_1_	{*main*, *comm*}
*C*_2_	{*io*, *publish*}
*C*_3_	{*filter*, *plan*}
*C*_4_	{*control*, *exec*}

**TABLE 7:** WCRT of HRT tasks considering the new affinity (Table 6).

HRT task	WCRT (ms)
main	0.98
comm	0.98
io	0.98
filter	0.95
control	0.92

### 5.2 Step Two

We generate, from the affinity in Table 6, the number of cores, and the GenoM3 system, an UPPAAL-SMC model. In the latter, schedulability for HRT tasks (Section 9.3) is guaranteed by construction (step one).

Now, using UPPAAL-SMC, we guarantee, up to a high probability, that low-priority tasks never starve, a less critical, yet important property (Section 3.1). To do so, we reason as follows. We know that, in any task manager (Figure 3), location *manage* denotes that a hyperjob is being executed. Thus, the absence of starvation means that (i) location *manage* is reachable and (ii) whenever it is reached, location *manage* is eventually left (back to location *start*). (i) is a reachability property, while (ii) is a *leadsto* (special type of liveness) property which UPPAAL-SMC does not support. This is a limitation of the tool and not intrinsic to SMC.

Fortunately, there is a simple workaround if we augment the manager model (Figure 3) with a clock *x* that is reset to 0 whenever any location is left. Thus, if the value of *x* is upper-bounded, then *manage* (i) is reachable (otherwise *x* would be unbounded at *start* or *ask*) and (ii) is eventually left (otherwise *x* would be unbounded at *manage*), which correspond to the same (i) and (ii) above. We may thus transform the two-step reachability–leadsto property into a safety property as we query the UPPAAL-SMC verifier to estimate the probability of *x* being bounded by value *x*_ max, e.g., for task plan: Pr[ ≤ *b*]([]*manager*_*plan*.*x* ≤ *x*_ max) (with *b* being a time bound for SMC simulations). We call *prob*_*t*_ the probability of satisfying this property by a task *t*.

We set the statistical parameters to a high confidence (*α* = 0.02) and precision (*ϵ* = 0.002), which means that the highest probability we can obtain for *prob*_*t*_ is 99.8*%* ±*ϵ*, *i.e.*, *prob*_*t*_ ∈ [0.996, 1] with a confidence 100*%* − *α* = 98*%*. For each task *t*, we set *x*_ max to *P*_*t*_ and raise it until such highest probability is reached.

Table 8 gives the results for all low-priority tasks: each is starvation-free with a *99*.*8%* probability as soon as *x*_*max* = 7 ms. This means that, for any value smaller than 7 ms, some low-priority tasks have a very low probability to complete given any execution scenario. For instance, as shown in Table 8, the probability that task exec will always execute in less than *x*_*max* = 6 ms is comprised between 0 and 4 percent, a value that increases significantly to the maximum possible probability given the precision (99.8*%* ± 2*%*) when *x*_*max* is increased to 7 ms. In sum, we have a high confidence that the time separating the activation and the end of execution of any low-priority task is upper-bounded by the value 7 ms.

**TABLE 8:** Verification results (step two).

	*prob*_*t*_ ∈			
***t***	***x*_max_ = 4**	***x*_max_ = 5**	***x*_max_ = 6**	***x*_max_ = 7**
publish	[0, 0.004]	[0, 0.004]	[0.996, 1]	[0.996, 1]
plan	[0, 0.004]	[0, 0.004]	[0.996, 1]	[0.996, 1]
exec	[0, 0.004]	[0, 0.004]	[0, 0.004]	[0.996, 1]

UPPAAL-SMC takes up to 25 min to verify each property, a value that grows exponentially if we try to tighten the precision further: with *ϵ* tending toward zero, SMC tends toward classical model checking, and thus, scalability is threatened as we noticed in Foughali et al. (2019b).

### 5.3 Discussion

We prove, with certainty, the schedulability for all HRT tasks in the application while proposing a scheduling policy on the drone platform. Also, we prove with a high probability that low-priority tasks never starve for cores. Thus, considering the real robotic platform and the affinity and scheduling algorithm we propose, the GenoM3 system of the drone guarantees the latter does not crash because HRT constraints are not met and is highly likely to fulfill its navigation missions (Section 3.1).

However, schedulability tests of HRT tasks barely pass (the WCRT of each task is quite close to its deadline, Table 7). This means that, in reality, tasks may still miss their deadlines due to, e.g., the overhead induced by the global MSRP implementation. Since the overheads of locking protocols are hard to quantify and upper bound in the general case, it would be better if tasks’ WCRTs were significantly smaller than their respective deadlines. Also, though a lower priority task missing its deadline is not safety critical (i.e., will not lead to a crash), it is still *mission critical* (will likely cause a larger time for the drone to fulfill a navigation mission). These results may be enhanced by replacing global MSRP with a fine-grained locking protocol to tighten the blocking bounds (due to spinning for resources) and improve schedulability, as we will see in the rest of this paper.

## 6 Setting the Requirements for Real-Time Locking in Robotics

As we have explained in Section 2 and Section 4, the locking model used in GenoM3 is not exempt of starvation and not necessarily fair, which makes it *unpredictable*. In Section 4 and Section 5, we proposed to use a predictable (fair and starvation-free) global locking protocol, namely, global MSRP. As we explained in the same sections, global MSRP introduces larger blocking bounds because all resources are locked at once, making TU codels wait for other TU codels that do not necessarily use the same set of resources. What we need is a *fine-grained* real-time locking protocol that is predictable, suitable for robotics, and, ideally, *efficient* (low overheads). Before we set more precisely the requirements for such needed protocol, we first formalize a generic real-time model for GenoM3 systems (based on the one given in Section 4) including a formalization of the locking model of GenoM3 explained informally in Section 2.

### 6.1 Real-Time Model

From a real-time point of view, a GenoM3 system is made of a set of dependent tasks, a set of shared resources, and a set of cores.

#### 6.1.1 Task Model

The set of tasks is *τ* = {*t*_1_…*t*_|*τ*|_}, where |*τ*| is the number of tasks in *all components*. Each task *t*_*i*_ is defined as a set of *jobs* $t_{i}=\left\{J_{1}^{i}\ldots J_{\left|t_{i}\right|}^{i}\right\}$ , where each job (called hyperjob earlier) $J_{k}^{i}$ is an *ordered* set of critical sections $J_{k}^{i}=\left\{cs_{k,1}^{i}\ldots cs_{k,|J_{k}^{i}|}^{i}\right\}$ , with each critical section being simply a codel. Contrary to the model introduced in Section 4, we refer to hyperjobs simply as jobs and use the subscript of a task as superscripts in jobs and critical sections belonging to such a task (as before, superscripts/subscripts are omitted when unnecessary, unimportant, or clear in the context). We may thus obtain $J=\cup_{i\in 1‥|\tau |}\left(\cup_{k\in 1‥|t_{i}|}J_{k}^{i}\right)$ and $\mathrm{CS}=\cup_{i\in 1‥|\tau |}\left(\cup_{k\in 1‥|t_{i}|}\left(\cup_{l\in 1‥|J_{k}^{i}|}cs_{k,l}^{i}\right)\right)$ , respectively, for the set of all jobs and all critical sections in the system.

The same notations as in Section 4 are used for task periods and priorities and WCETs of critical sections (codels). The set of shared resources is *L* = {*l*_1_…*l*_|*L*|_}. The function R:CS↦P(L) (where P(L) is the power set of *L*) associates each critical section with *all the resources* it needs for its execution, regardless of the mode (read-only or write mode, Section 2) in which such resources are accessed. Finally, the set of cores is $C=\left\{C_{1}\ldots C_{\left|C\right|}\right\}$ .

#### 6.1.2 Behavior

Except for a more complex notion of jobs, the above model is essentially equivalent to the generic sporadic task model (Brandenburg, 2011). However, the behavior is constrained by two specificities of the robotic context (regardless of the used scheduler): (i) *spinning* is favored over *suspension* and (ii) preemption is disallowed during both spinning and execution of a critical section and may thus be viewed as a kind of limited preemption model (Buttazzo et al., 2012).

At each period $P_{t_{i}}$ , task *t*_*i*_ is *activated*. When *released*, *t*_*i*_ executes job $J_{m}^{i}$ (chosen at runtime) by sequentially executing its ordered set of critical sections $cs_{m,1}^{i}\ldots cs_{m,|J_{m}^{i}|}^{i}$ , where each critical section *cs* is executed iff no other critical section *cs*′ that is *in conflict* with *cs* (see below) is being executed; otherwise, *t*_*i*_ spins non-preemptively. *t*_*i*_ terminates when the execution of $J_{m}^{i}$ ends, i.e., when it finishes executing $cs_{m,|J_{m}^{i}|}^{i}$ , the last critical section in $J_{m}^{i}$ . If the scheduler is preemptive, preemption is allowed only between critical sections: regardless of its priority, *t*_*i*_ is non-preemptible from the moment it starts spinning or executing a critical section *cs* to the moment it finishes executing *cs*.

#### 6.1.3 Resource Conflicts

Locking in GenoM3 is handled at the critical sections’ level using statically defined *conflicts*. To formalize the model given in Section 2, we first introduce a new function $R_{w}:\mathrm{CS}\mapsto P\left(L\right)$ that returns for each critical section *cs* the set of resources that *cs* accesses exclusively in write mode. Therefore, *R*_*w*_(*cs*) ⊆ *R*(*cs*), and *R*_*r*_(*cs*) = *R*(*cs*) \ *R*_*w*_(*cs*) is the set of resources accessed by *cs* in read-only mode. Accordingly, the locking model of GenoM3 marks two critical sections *cs* and *cs*′ (in two different tasks) as *in conflict* iff there is at least one resource used by *both* *cs* and *cs*′ that is accessed in write mode by *cs* or *cs*′, i.e., either the intersection *R*(*cs*) ∩ *R*_*w*_(*cs*′) (between all resources used by *cs* and resources used by *cs*′ in write mode) or the intersection *R*(*cs*′) ∩ *R*_*w*_(*cs*) is not empty. Formally,

*cs* and *cs*′ are in conflict iff (*R*(*cs*) ∩ *R*_*w*_(*cs*′)) ∪ (*R*_*w*_(*cs*) ∩ *R*(*cs*′)) ≠ ∅ (Equivalence 1).

Note how this model is *multi-resource* and *nesting-free* in accordance with the “elementary code” design in robotics (Section 10). This locking model is also reader/writer. However, this model is *underspecified* and unpredictable, as neither fairness nor starvation freedom is guaranteed (though deadlock freedom is).

The real-time model given in this section covers all we need for real-time analysis. Therefore, whenever possible, we will drop terms such as codels and services and stick to the notation of this real-time model.

### 6.2 Requirements

Following the observations made in Section 2 and the real-time model above, we define a set of requirements *w.r.t.* the real-time locking implementation needed in robotics. We recall that the objective is to devise a predictable and efficient fine-grained implementation which will allow us to obtain shorter blocking bounds and thus improve schedulability and other real-time properties (Section 5.3).

Let us first summarize our observations:

• Locking in robotic software suffers from predictability and/or efficiency issues (this is not the case for GenoM3 only, more in Section 10).
• Resources are typically accessed in a multi-resource fashion (lock and unlock at once all the resources needed by a critical section).
• Multi-resource locking in robotics can be refined using knowledge on the mode each resource is accessed in by a critical section (reader/writer locking), where multiple resources in different modes may be locked simultaneously (*mixed read–write*).
• Typically, critical sections have short WCET, which explains in part favoring spinning over suspension.
• The number of resources is relatively large (e.g., over 30 in the drone application, Section 2.2).
• The number of cores is small due to SWaP considerations (Section 1.1), e.g., four in the drone application (Section 2.2).

Thus, what we need is a real-time locking implementation that is as follows:

• Multi-resource, nesting-free, reader/writer (mixed read–write).
• Predictable: fair and starvation-free, ideally with the smallest blocking bounds possible (e.g., *asymptotically optimal* blocking bounds).
• Efficient in the context of small number of cores and large number of resources: low overheads.

We analyze below existing state-of-the-art multi-resource locks and show why we need a new implementation to comply with all the requirements above. Such new implementation is then presented in the next section (Section 7).

### 6.3 Analysis of Existing Multi-Resource Locks

Multi-resource locking protocols acquire exclusive ownership of *multiple* resources *R*(*cs*) in a *single* request operation and conversely release these resources in a single operation as well. To comply with the requirements above, we assume non-preemptive execution during a critical section and spinning.

We assume that an implementation of a multi-resource locking protocol uses *resource bitmasks* as representation for *R*(*cs*), i.e., individual resources are denoted by a bit in an array of integers. All discussed multi-resource locking mechanisms expose such an interface.

One way to implement a multi-resource locking mechanism is a multi-bit *test and test-and-set* (TATAS) lock. Each bit in a machine word^9^ represents one resource, and a lock operation succeeds if all bits of requested resources can be changed from 0 to 1 atomically. However, TATAS locks do not support any ordering of concurrent requests, thus showing the risk of starvation and unbounded spinning.

Considering FIFO ordering of concurrent resource requests for *fairness*, two fundamentally different approaches can be used by a locking protocol: (i) Use *one* FIFO queue to order all resource requests. A later resource request is blocked by earlier conflicting request until all earlier conflicting resource requests have released their resources. Alternatively, (ii) use *multiple* per-resource FIFO queues, one for each resource, and acquire the requested resources in a *nested* fashion and in total order. A request to multiple requested resources is granted when all individual nested requests succeed. Still, both approaches have exactly the same blocking bounds, if we neglect implementation overheads.

This duality between multi-resource locks and nested locking allows the real-time nesting locking protocol (RNLP) family (Ward and Anderson, 2012; Ward and Anderson, 2013; Ward and Anderson, 2014; Ward, 2016) to provide a solution in both use cases. Dynamic group locks (DGLs) (Ward and Anderson, 2013; Ward, 2016) are the multi-resource lock variant of RNLP, and reader–writer RNLP (R/W RNLP) (Ward and Anderson, 2014) provides a reader–writer extension to DGL. With contention-sensitive RNLP (C-RNLP) (Jarrett et al., 2015), there is also an extension to RNLP that relaxes the strict FIFO ordering and tries to dynamically eliminate *transitive blocking chains*. The RNLP family provides the tightest blocking bounds known in the real-time literature, proven to be asymptotically optimal.

Conceptually, RNLP locks are always presented by using dedicated queues per resource in the literature (Ward and Anderson, 2012; Ward and Anderson, 2013; Ward and Anderson, 2014; Ward, 2016). However, later work of the authors gives a hint to single-queue implementations of the non-reader–writer variants (Jarrett et al., 2015). R/W RNLP cannot be implemented using a single queue, as its complex arbitration rules require multiple queues.

All RNLP implementations have a similar structure. One or more internal locks protect internal state (one or many queues), and the locks must be taken in both request and release operations. Also, there is a Boolean blocking condition *outside* any internal critical sections where a lock request operation performs busy-waiting on resource conflicts.

Another notable multi-resource is Zhang et al.’s *MRLock*, based on a single *lock-less* queue that tracks all resource requests in FIFO order (Zhang et al., 2013). Requests can comprise an arbitrary number of resources, and insertion into and removal from the queue happen in a lock-free manner. MRLock is thus conceptually similar to single-queue implementations of DGL but replaces the lock-based queue by a lock-less one, making it remarkably efficient (as it eliminates the overheads of locking and unlocking the queue itself). However, MRLock is designed for best-effort use cases without real-time scenarios in mind, e.g., preemptive high-performance computing. Therefore, its design tolerates preemption of lock or unlock operations at any time. This makes MRLock unsuitable for real-time applications, as it suffers from a degraded predictability in corner cases. In particular, MRLock loses its fairness and/or starvation freedom when it reaches the limits of its internal queue, e.g., when too many new short-running requests arrive and complete, but previous older long-running requests are still busy. This drawback may be viewed as a structural side-effect of the lock-less queue (Zhang et al., 2013, Section 4.2).

Multi-resource locks can be extended to reader–writer multi-resource locks to further tighten the blocking bounds. For this, we distinguish between resources requested for shared read access *R*_*r*_(*cs*) and resources requested for exclusive write access *R*_*w*_(*cs*) and require that an implementation provides an interface to specify both *R*_*r*_(*cs*) and *R*_*w*_(*cs*) in lock and unlock operations.

The precedence by FIFO ordering works well for exclusive multi-resource locks and results in *fair* ordering of all requests. However, when extending exclusive multi-resource locks to reader–writer locks, we must also consider the ordering of read and write requests to each other. Note that the standard reader-preferring and writer-preferring reader–writer lock variants are unsuitable for real-time systems, as they starve either writers or readers.

*Task-fair* reader–writer locks order arriving requests in FIFO order but allow adjacent read requests to form a *concurrent group* until the next write request arrives (Mellor-Crummey and Scott, 1991b). Note that strictly alternating read and write requests define the worst case for task-fair reader–writer locks where the locks degrade to fair non-reader–writer locks in behavior. Task fairness emerges automatically if an implementation follows the formalization of conflicts in Section 6.1.3 and ensures FIFO ordering of all requests.

Another mechanism suitable for real-time systems is *phase fairness* (Brandenburg and Anderson, 2010), where requests are queued in either read or write request queues, and reader and writer phases alternate. Then, on a phase switch to readers, all waiting readers are released. This improves the throughput of read requests at the cost of write requests.

To the best of our knowledge, R/W RNLP and *fast* R/W RNLP are the only real-time reader–writer multi-resource locks described in the literature (Ward and Anderson, 2014; Nemitz et al., 2019a). R/W RNLP provides *phase fairness*. Its extension *fast* R/W RNLP distinguishes between nested and non-nested requests and provides a fast-path for non-nested requests, which request only a single resource. However, R/W RNLP and *fast* R/W RNLP provide an interface with explicit lock and unlock operations for *R*_*r*_(*cs*) and explicit lock and unlock operations for *R*_*w*_(*cs*), but not a *combined* interface that allows to specify both *R*_*r*_(*cs*) and *R*_*w*_(*cs*) at the *same* time. The authors discuss the possibility of such a “mixed mode” interface but provide no implementation (Ward and Anderson, 2014). We assume that an implementation, if possible, would be non-trivial due to the overall complexity of the entitlement mechanisms. Hence, both R/W RNLP and *fast* R/W RNLP are useless for mixed read–write requests. We skip the *fast* R/W RNLP in the remainder of this work, as the fast-path to request a single resource does not help in the robotic use case.

Opposing the requirements presented in Section 6.2 to the analysis made in this section leads to the following conclusion. Predictable (with asymptotically optimal blocking bounds) multi-resource real-time locking protocols exist, namely, DGL, but no suitable reader–writer variant is available for mixed read–write needed in robotics. Also, DGL efficiency may be improved through the use of a lock-less structure, such as in MRLock, to eliminate internal overheads. Therefore, in the next section, we will present an implementation that exactly tackles these two limitations. Indeed, our R/W LLAB implementation features the predictability of DGL with the same asymptotically optimal bounds (and better blocking bounds in practice because of its reader/writer nature). At the same time, it (i) is suitable for mixed read–write requests and (ii) has lower overheads than DGL as it uses a lock-less implementation, as we will see throughout Section 7 and Section 8.

## 7 Lock-Less Array-Based Multi-Resource Reader–Writer Locks

We present an efficient-and-predictable multi-resource lock that supports task-fair reader/writer locking with mixed read–write requests.

The analysis in Section 6 shows different design techniques for queuing and internal locking. First, we stick to a design of using resource bitmasks, like in the other approaches. This helps to handle a large number of resources. Second, we opt for a single-queue design that orders all requests logically in FIFO order. The single-queue approach allows to use simple checks if older requests on the queue are in conflict with newer requests, and the number of requests on the queue is bounded by the number of CPU cores. Also, a check for conflict based on resource bitmasks is agnostic to the actual number of requested resources. Third, we aim for a lock-less design. But instead of using a lock-less queue as in MRLock, we use a design based on a fixed-size array (number of CPU cores) where a core’s ID relates to the index in the array. We then establish an FIFO order on the requests by using a ticket mechanism, similar to ticket spinlocks. This allows to use the relative difference of drawn tickets to distinguish newer from older requests. Also note that checks for conflicts do not exactly need to happen in the FIFO order. A request just needs to ensure to visit all older requests on the queue for correctness. For efficiency, we iterate the fixed-size array in order, identify older requests based on the relative age of their ticket, and then check and spin on conflicting requests. Lastly, we must handle race conditions that can happen on concurrent insertion of requests. We aim for a design that shows a low overhead for non-conflicting but concurrent requests.

### 7.1 Task-Fair Conflict Check for Multi-Resource Reader–Writer Locks

Task-fair reader–writer locking follows the rules explained in Section 6.1.3. To alleviate notations, we use an abuse of terminology, as we let *R*(*cs*) denote both the set of requested resources and the request itself preceding the execution of critical section *cs*. We also extend the term “conflict” to include sets of resources as well (two sets of resources *R*(*cs*) and *R*(*cs*′) are in conflict if critical sections *cs* and *cs*′ are in conflict, Section 6.1.3). Therefore, a lock request *R*(*cs*) = *R*_*r*_(*cs*) ∪ *R*_*w*_(*cs*) (*R*_*r*_(*cs*) for reading and *R*_*w*_(*cs*) for writing) made by a task *t* (to execute critical section *cs*) is in conflict with an older request *R*(*cs*′) = *R*_*r*_(*cs*′) ∪ *R*_*w*_(*cs*′) made by task *t*′ (to execute critical section *cs*′) iff Equivalence 1 is satisfied (Section 6.1.3). From an implementation point of view, Equivalence 1 uses the corresponding bitmasks to both *R*(*cs*) and *R*(*cs*′) in the conflict check. Since these bitmasks include bits of all resources requested by *cs* and *cs*′, it is desirable to use them the least possible in the checks. We may therefore redefine Equivalence 1 to include only *R*(*cs*′) as follows:

*cs* and *cs*′ are in conflict iff (*R*_*r*_(*cs*) ∩ *R*_*w*_(*cs*′)) ∪ (*R*_*w*_(*cs*) ∩ *R*_*w*_(*cs*′)) ∪ (*R*_*w*_(*cs*) ∩ *R*(*cs*′)) ≠ ∅ (by replacing *R*(*cs*) in Equivalence 1 with *R*_*r*_(*cs*) ∪ *R*_*w*_(*cs*) and then distributing union over intersection).

Then, we obtain the following:

*cs* and *cs*′ are in conflict iff (*R*_*r*_(*cs*) ∩ *R*_*w*_(*cs*′)) ∪ (*R*_*w*_(*cs*) ∩ *R*(*cs*′)) ≠ ∅ (Equivalence 2) (by getting rid of (*R*_*w*_(*cs*) ∩ *R*_*w*_(*cs*′)) since it is included in (*R*_*w*_(*cs*) ∩ *R*(*cs*′))).

We therefore store *R*(*cs*) = *R*_*r*_(*cs*) ∪ *R*_*w*_(*cs*) for each request in the array. Then, checking for the absence of conflicts (Equivalence 2) becomes a conjunction of two bitwise AND operations, each operating on each integer in two resource bitmasks (see the explanation of the listing below).

### 7.2 Implementation

Listing 2 shows the implementation of the lock-less array-based (LLAB) multi-resource lock with task-fair reader–writer locking, named “R/W LLAB,” in C language. The presented implementation is suitable for architectures with a relaxed memory model, such as ARM. Atomic load/store operations are annotated with *relaxed*, *acquire*, or *release* semantics following the C11/C++11 memory model but must be adapted to the OS or runtime environment. For brevity, the presented version uses 64-bit integers for resource bitmasks. The non-reader–writer variant LLAB can be derived from R/W LLAB by assuming that all resources are write requests. A commented and extended version of Listing 2 can be found in our git repository^10^. We discuss the implementation below.

With the number of cores known at compile time (line 1), the FIFO queue is implemented as an array of nodes statically assigned to cores in a one-to-one exclusive mapping (line 12). Each node comprises a *drawn ticket* and two bitmasks of resources for reading and writing (lines 5 to 8). Bitmasks of known size (line 3) track the requested resources, and tickets ensure FIFO ordering of tasks through drawing from a *global ticket counter*. The global data of the lock object comprise thus a global ticket counter and the array of nodes (lines 10 to 13).

Listing 2:
Implementation of R/W LLAB.

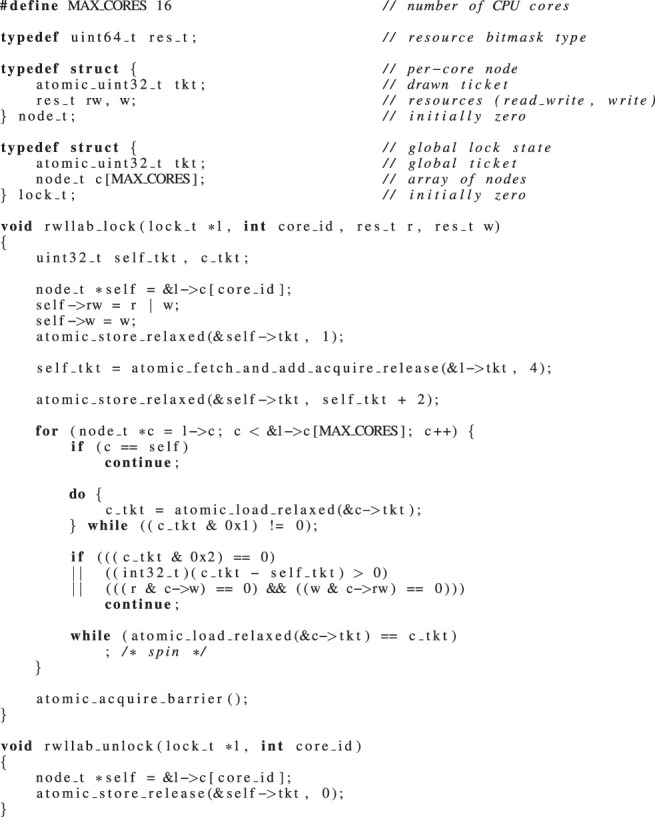

To address race conditions on insertion, the implementation uses two least significant bits of the drawn ticket recorded in the array node of core *C* to capture the status of the request:

• If *ticket* mod 4 = 0, then the request by core *C* is *inactive*, i.e., no task is currently trying to execute, or a task just finished executing a critical section on core *C*,
• if *ticket* mod 4 = 1, then the request by core *C* is *in preparation* (see below), and
• if *ticket* mod 4 = 2, then the request by core *C* is *active*, i.e., a task is executing or spinning to execute a critical section on core *C*.

Accordingly, the global ticket is always incremented by 4 (line 24) to keep the two least significant bits free.

The basic workflow of a lock operation is as follows: (i) prepare a request (lines 19 to 21), (ii) set the drawn ticket number to 1 to indicate the request as *in preparation* (line 22), (iii) draw a unique ticket (line 24), (iv) store the drawn ticket in *C*’s node with the *active* bit set (line 26) and then (v) iterate all other cores’ requests (lines 28 to 43), (vi) spin on requests in *in preparation* state (lines 32 to 34), (vii) check for potential conflicts (lines 36 to 39), and (viii) spin on resource conflicts (lines 41 to 42).

Note that the lock operation comprises two different spinning points. In step (vi), spinning on requests in *in preparation* state if the ticket counter is 1 (lines 32 to 34) ensures correctness in case a race condition happens when a lock operation is delayed between steps (iii) and (iv), e.g., by an interrupt, as drawing a ticket (line 24) and storing the ticket and thus marking the request as *active* (line 26) is not an atomic operation. This ensures that the later checks in step (vii) correctly observe the drawn ticket of that request. The second spinning happens on actual resource conflicts (lines 41 to 42). But first, the lock operation checks that the request of another one is *active* (line 36), that this request is older than its own request (lower relative ticket number) (line 37), and that the request is in conflict (resource bitmasks conflict) (line 38). If all conditions are met, the lock operation then spins until the requests’ ticket number changes. The *unlock* operation simply sets the ticket number to zero (line 51), which marks its former request as *inactive*.

In the following, we provide some important implementation details that are however not necessary to understand the behavior described above.

First, ordering memory accesses is crucial in this algorithm. Setting a node to *in preparation* state (line 22) must become visible to the other cores *before* drawing a new ticket, i.e., before the store of the *fetch-and-add* completes (line 24), as the algorithm explicitly spins on this state (lines 32 to 34), and the store of the drawn ticket in the node (line 26) must happen afterward. Likewise, the requested resources of a node (lines 20 and 21) must be visible *before* checking them (line 38). Both properties are ensured by drawing the ticket atomically with *acquire* and *release* semantics (line 24). This operation enforces a global ordering of the three steps (ii) to (iv) of the algorithm and allows to use *relaxed* semantics before and afterward. Note that this ordering between a node’s resource bitmask and its ticket value is only guaranteed for resource requests that happened *before* drawing the ticket, i.e., all *previous* resource requests, but not for newer requests that happen later. However, the algorithm does not depend on an exact ordering for newer requests, as it skips newer nodes solely on their ticket number. The final *acquire barrier* in the *lock* operation (line 45) pairs with the *store release* in the *unlock* operation (line 51). Note also that spinning for changes of the ticket value (lines 41 and 42) using *acquire* semantics would not be a sufficient replacement for the final *acquire barrier* (line 45), as other cores might release the shared resources (line 51) briefly before the check of the ticket state (lines 36 and 37).

Second, the global ticket counter can overflow; therefore, the implementation must check the *relative age* of drawn ticket numbers (line 37). Additionally, for any ticket number that is recorded in a node, the related critical section must complete before the same ticket number is withdrawn again. A 32-bit counter incremented by 4 provides 2^30^ unique tickets, and the relative age changes sign after 2^29^ unique tickets, so recurring tickets are not a problem on real systems.

Our design for LLAB and R/W LLAB draws on techniques found in ticket locks (Mellor-Crummey and Scott, 1991a) to establish an FIFO order between requests and on the idea to wait on other nodes to complete internal operations found in the unlock path of MCS locks (Mellor-Crummey and Scott, 1991a). The idea to encode additional state in the ticket counter can also be found in phase-fair reader–writer locks (Brandenburg and Anderson, 2010).

Conceptually, LLAB and R/W LLAB behave exactly as the single-queue implementation of DGL, thus preserving the same theoretical blocking bounds, but come with two additional advantages. First, their lock-less structure removes the internal overheads found in DGL, making them more efficient. Second, R/W LLAB is reader/writer and supports mixed read–write requests, thus tightening further the blocking bounds by allowing some simultaneous readings (Section 8). To the best of our knowledge, R/W LLAB is the first real-time multi-resource locking protocol that supports mixed read–write requests.

## 8 Evaluation

We perform two different types of experiments to evaluate LLAB and R/W LLAB among the other multi-resource locks. The first type evaluates any internal overheads in the uncontended case (Section 8.1), where we use two hardware platforms: an ARM system with four cores (Raspberry Pi 2B running an RTOS) and an Intel system with 2x 16 cores/64 hardware threads (2x Intel Xeon Silver 4216 running Linux). The ARM system is representative of real-time robots, where MECs feature a low number of cores (Section 6.2), whereas the Intel system allows for an evaluation in a more generic multi-core setting. The second type of experiment measures the overheads of an execution scenario with mixed reader–writer workloads using randomized critical sections on the ARM platform (Section 8.2). In each experiment, we compare the presented locking mechanism LLAB resp. R/W LLAB with other multi-resource locks, namely, DGL (Ward and Anderson, 2013), R/W DGL (our task-fair reader–writer variant of DGL), R/W RNLP (Ward and Anderson, 2014), MRLock (Zhang et al., 2013), and a multi-bit *test and test-and-set* (TATAS) lock. The implementation of DGL uses a single queue and is obtained from Nemitz et al. (2019a). A fast-path optimization allows non-conflicting requests to bypass the queue^11^. R/W DGL is our own task-fair reader–writer extension of DGL. It is based on a single queue as well and uses the conflict check described in Section 7.1. But due to the nature of reader–writer locks, it cannot use the fast-path to bypass the queue^12^. Our implementation of R/W RNLP that uses phase fairness follows the pseudocode from Ward and Anderson (2014). As resources are managed in dedicated queues and the resource bitmasks in our benchmark are typically only sparsely populated, we use an efficient iterator for bitmasks (see Table 9). A naive implementation of a for-each-bit operation based on a loop over all bits in a machine word causes too much overheads. Fortunately, modern processor architectures provide instructions to count leading or trailing zeros in machine words, so we use compiler intrinsics such as GCC’s __builtin_ctz() for efficient iteration. Any internal locking in our experiments is based on MCS locks (Mellor-Crummey and Scott, 1991a) and phase-fair reader–writer ticket locks (Brandenburg and Anderson, 2010). For MRLock and TATAS, we use the example code from Zhang et al. (2013). We instantiate MRLock with an internal queue of 256 nodes (*cells* ^13^) on Intel and 16 nodes on ARM.

**TABLE 9:** Average execution time overhead (CPU cycles) of different iterators and locks in isolation.

Test or lock	Resources	ARM	Intel
CPU cycles in 1*μ*s		600	2400
Naive bit iterator	1	537	151
(Bitwise shift and &)	64	594	93
Efficient bit iterator	1	11	5
(__builtin_ctz())	64	342	193
TATAS	1..64	87	37
MRLock	1..64	172	51
DGL	1..64	278	67
R/W DGL	1..64	290	66
R/W RNLP read lock	1..64	166	35
Write lock	1	649	196
	64	3713	857
LLAB	1..64	136	199
R/W LLAB	1..64	154	224

We added memory barriers with *load-acquire* or *store-release* semantics where needed for the weak memory model on ARM and pause instructions on x86 to yield to other hardware threads when spinning. Locks and core-specific internal data are aligned to cachelines to prevent false sharing. All lock types support up to 64 resources, i.e., resource bitmasks are 64-bit sized. We provide implementations of all locks in our git repository.

### 8.1 Internal Overheads in the Uncontended Case

**FIGURE 5:**
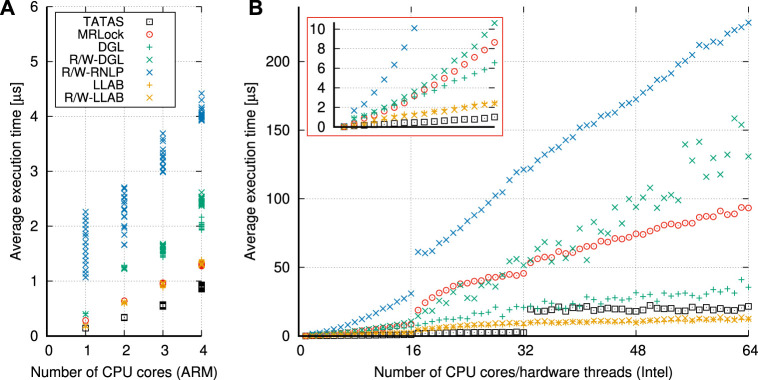
Average execution time of a *lock* → *unlock* sequence for a variable number of uncontended resources on different hardware architectures in parallel. Each lock type supports 64 resources. **(A)** shows results for ARM Cortex-A7 at 600 MHz with four physical cores. The stacked data points represent the results for an increasing number of requested resources, i.e., from 1 to a limit of 16 resources per core. **(B)** shows results for dual Intel Xeon Silver 4216 at 2.1 GHz. Here, cores 1 to 16 are physical cores on the first CPU, and cores 17 to 32 are physical cores on the second CPU. Cores 33 to 48 are hardware thread siblings of the cores on the first CPU and cores 49 to 64 of the cores on the second CPU. Each core requests one resource related to its core ID. The red box zooms in on the results of the first 16 cores.

In the first experiment, we measure the overhead in the uncontended case. For this, we let a thread lock and unlock an increasing number of exclusive (i.e., write) resources in a tight loop and measure the execution time of 1024 lock → unlock sequences. This shows the performance impact of the *number of requested resources* in a lock or unlock request as vertically stacked data points for the different types of locks. The results show the average execution time of a single lock → unlock sequence, including any outliers. Additionally, we run this test on a number of cores in parallel. Each core locks and unlocks resources private to the core (so they are uncontended), but in the same shared lock. This exposes the overhead of any *internal* synchronization (e.g., internal locks or CAS-loops) of the different locking mechanisms. Figure 5A shows the results for the ARM system and Figure 5B for the Intel system. As the data points on a single core are next to each other, Table 9 shows the results on a single core in detail for both architectures.

At first, it becomes visible in Figure 5A that R/W RNLP shows an execution time that is *sensitive* to the number of requested resources in each lock → unlock sequence, during which the lock implementation iterates the resources three times. This is easily explained by the design choice in R/W RNLP to use dedicated queues per resource. In contrast, TATAS, MRLock, DGL, R/W DGL, LLAB, and R/W LLAB are *insensitive* to the number of requests. It also becomes visible that R/W RNLP is exceptionally slow compared to the rest. This is mostly due to the internal locking overhead. The R/W RNLP lock operation comprises three internal critical sections, adding much static overhead.

On a single core and with just a few resources, the performance of all locks is next to each other, as shown in Table 9. But with an increasing number of cores, internal overhead increases and scalability decreases. On Intel, for a larger number of cores until the number of physical cores on the first CPU (16) is reached (red box in Figure 5B), TATAS scales best, immediately followed by LLAB and R/W LLAB; then, DGL, MRLock, and R/W DGL follow next with already more than twice the execution time when using 16 cores. The trend amplifies when crossing the boundary to the second CPU from core 17 on. Beyond the 32 available physical cores, the Linux kernel starts to schedule the tasks on each core’s hardware thread sibling as well. Then, the performance of TATAS drops significantly. LLAB and R/W LLAB become the fastest lock implementations, followed by TATAS and DGL. With a large gap, MRLock and R/W DGL follow. In all cases, R/W RNLP is the lock with the most overhead. The performance of DGL shows that it runs in fast-path mode with an empty queue. Its performance characteristics relate to two consecutive MCS lock → unlock operations. R/W DGL and MRLock additionally need to search the queue for conflicts. We were surprised that LLAB scales better than MRLock on the Intel system, as our initial results on ARM showed that both were next to each other. We assume that the extra overheads in MRLock are caused by reading head and tail pointers to queue nodes in each internal loop, while LLAB and R/W LLAB scan their queues linearly from front to back. With this, MRLock shows one additional level of pointer indirection than R/W LLAB. Also, the memory layout of LLAB and R/W LLAB is more compact than that for MRLock.

Overall, LLAB and R/W LLAB have a visible performance advantage (as one can see from Figure 5), which matches our expectations of Section 7.

### 8.2 Mixed Reader–Writer Workloads

As a second benchmark, we evaluate the locks in an experiment using randomly generated critical sections with synthetic mixed reader–writer workloads similar to the case study presented in Section 2.2. On the ARM system with four cores, we run a task set with one periodic task per core (period 1 ms). Each task locks and unlocks a random number of critical sections (1 to 8, following a square distribution) comprising randomized read and write requests for up to 32 shared resources. The critical sections comprise 4.1 read and 1.9 write requests on average. The time spent in the critical sections follows a power function (1 to 17 *μ*s), but mostly favoring short critical sections. Our selected parameters and their distribution follow the execution scenario of the real drone example in Foughali (2020) and our observations on other GenoM3 systems such as the autonomous terrestrial robot used in Foughali et al. (2020). For instance, our quad-core ARM system is similar to the ARM-based quad-core ODROID, and the critical sections’ execution times are upper-bounded with the WCETs of the critical sections in Foughali (2020).

**FIGURE 6:**
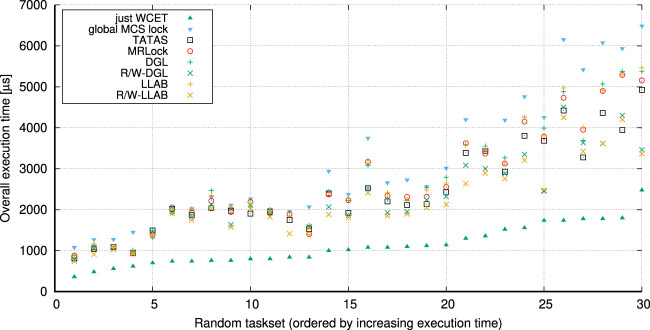
Synthetic mixed reader–writer workload scenario with four periodic tasks executing in parallel on ARM using a randomized set of critical sections requesting multiple resources. The dataset “just WCET” shows the nominal execution time inside the critical sections (without blocking). The dataset “global lock” shows the overhead when using a global lock instead of multi-resource locks.

To remove any differences in the tasks’ release times introduced by the hardware or the operating system, we let the tasks synchronize at each period on a barrier before executing the critical sections and then add a random release jitter of up to 0.1 *μ*s. We then measure the execution time from before acquiring the first critical section to after releasing the last critical section and then sum the execution times of all tasks on all cores for 20 periods (20 ms) into a single score. Figure 6 shows the results for different lock types in 30 generated task sets. As references, we also show the accumulated execution time inside the critical sections (minimum possible execution time without any blocking, “just WCET”) as well as a run using global MSRP implemented with an MCS lock (maximum blocking time) as lower and upper bounds. Here, we did not include R/W RNLP, as it provides no interface to lock both reader and writer requests at the same time (more in Section 8.3). This leaves R/W DGL and R/W LLAB as the only reader–writer locks. All other locks handle read requests as exclusive requests.

The results for short task sets with a low number of critical sections are next to each other. But for more complex task sets, three trends emerge. First, the generated critical sections favor the reader–writer locks R/W DGL and R/W LLAB over exclusive multi-resource locks. This hints the potential benefits of using reader–writer locks in such scenarios. Second, TATAS is one of the fastest locks. We expected this due to the simplicity of TATAS and the low number of resources. But note that TATAS locks do not guarantee FIFO queuing of conflicting requests. Third and lastly, the lock-less design of R/W LLAB beats the lock-based design of R/W DGL. But the other locks are next to each other without a clear winner. We also expected this, as there is not much internal contention on the lock due to the randomized time spent inside critical sections.

### 8.3 Discussion

Summarizing the benchmark results of Section 8, we can observe two major trends. First, reducing static overhead, e.g., the use of internal locks, matters for good performance. Second, the execution time of internal critical sections can be either *sensitive* to the number of requested resources, or *insensitive*. This sensitivity comes from the effect of using multiple queues, which requires iterating over resources instead of iterating over potential conflicts from other cores. We argue that insensitivity with its O(1) dependency on the number of resources and O(n) dependency on the number of conflicts scales better in the long term, as the number of resources usually grows faster than the number of cores that can cause conflicts. In a nutshell, when the number of cores is small and the number of resources is large, which is typically the case for real-time robots (Section 6.2), it is better to spin on conflicts rather than on resources. Our locks combine both trends (i) by spinning on conflicts instead of resources and (ii) by eliminating internal locks using a lock-less array (LLAB and R/W LLAB).

Compared to DGL, LLAB is predictable with exactly the same theoretical blocking bounds as DGL, but with lower overheads, as shown in our experiments. Furthermore, since conflicts may change at runtime, LLAB preserves the dynamicity of DGL, therefore making the locking in any application using the latter implementable in the former. Yet, DGL (and RNLP in general) also supports suspension, whereas LLAB is presented for a spin-based context.

Compared to R/W RNLP, our presented R/W LLAB provides *task fairness*, while R/W RNLP provides the better *phase fairness* (Brandenburg and Anderson, 2010). However, this is not visible in the evaluation, as R/W RNLP does not provide an interface to acquire mixed sets of read and write resources in the nesting-free multi-resource context, making R/W RNLP impractical to use in, e.g., robotic software. We assume that this is due to the structure of R/W RNLP that uses different types of internal locks for read and write requests to protect the internal state (Ward and Anderson, 2014).

It is worth mentioning that the way R/W LLAB works is somewhat similar to *concurrency groups* (Nemitz et al., 2019b). Yet, concurrency groups are computed offline, prior to the system execution, which makes them unsuitable for resource requests that may change from one execution to another. Lastly, we must note that DGL can be extended to a task-fair variant as well, as shown in our experiment with R/W DGL. In this case, R/W LLAB may be viewed as, essentially, a more efficient (lock-less) implementation of R/W DGL.

Compared to MRLock, both MRLock and LLAB map the single queue to an array. However, LLAB relies on non-preemptive locking in a real-time system for correctness, whereas MRLock also supports preemption at any time, which is inevitable in non-real-time best-effort systems. This adds additional complexity to the implementation, as the evaluation shows that LLAB scales better than MRLock on a larger number of cores. Additionally, MRLock has a different design of the array. While the array size in LLAB is limited by the number of available cores |*C*|, MRLock allows the user to configure a larger array to support more than |*C*| nodes at a time. When enqueuing requests, MRLock guarantees fairness as long as the array has free space. But when the next free node in the array is busy, e.g., because its lock-holding task was preempted, new requests start to spin for this task to finish and free its position in the queue. MRLock then loses its fairness guarantees (Zhang et al., 2013, Section 4.2). While this problem could be solved by selecting an array bound that is large enough to prevent this corner case, determining such bound may be hard for a real-time system using sporadic tasks. This is because we must at least ensure that the array is large enough so that the longest running critical section never collides with all possible combinations of shorter critical sections that run in the mean time.

As any locking algorithm, LLAB and R/W LLAB still have their drawbacks. One downside that does not become visible in the presented experiments is that the lock-less array design requires to scan the full array for potential conflicts. For a larger number of cores, the array-based designs must access a larger (constant) number of consecutive cachelines than the other lock implementations. For example, R/W LLAB uses 25 cachelines of 64 bytes on the Intel system with 64 cores/hardware threads. In contrast, the number of cachelines needed by the other lock implementation varies from 2 to 65, depending on the size of the queue, and because queue nodes are kept on distinct cachelines. However, the effect is practically invisible on systems with a small number of cores such as the ARM system with four cores that requires two cachelines to manage 64 resources with R/W LLAB, and thus, this downside is harmless in the case of real-time robots. Also, the linear memory accesses when iterating the array benefit from the prefetch units implemented in today’s CPUs.

To wrap up our discussion, the proposed LLAB and R/W LLAB fulfill our requirements (Section 6.2) for real-time robotic systems, or any other real-time system with non-preemptive critical sections as defined in Section 6.1. Our evaluation (Section 8) shows that LLAB, implemented as a lock-less array variant of DGL, excels in *efficiency* over the other solutions. Second, its task-fair reader–writer lock variant R/W LLAB addresses the use cases of robotic frameworks: it (i) is spin-based, multi-resource, nesting-free, and reader/writer with support for mixed read–write requests and (ii) is suitable for systems with a small number of cores and large number of resources. Third, LLAB and R/W LLAB use resource bitmasks, which may be updated at runtime, for both read and write requests, making them easier to use and less error prone than nested lock requests (no risk of deadlocks) and suitable for applications where conflicts between critical sections may change at runtime. Finally, both implementations have a simple configuration preventing any additional complexity as in MRLock. With this, LLAB and R/W LLAB can help improve the locking situation in today’s robotics frameworks (Section 2, Section 10). Also, they show promising scalability beyond the low number of cores found in real-time robots.

## 9 A Two-Step Hybrid Approach (Revisited)

As seen in Section 5, applying our two-step verification approach (Section 4) to the drone system (Section 2.2) gives acceptable results: all HRT tasks are schedulable and lower-priority tasks do not starve with upper-bounded execution times. However, as we point out in the discussion in Section 5.3, it is still desirable to have a model where such results correspond to better schedulability for both HRT and lower-priority tasks to account for the overheads of the locking protocol. Therefore, in this section, we assume that the concurrency in our drone system is handled using R/W LLAB, the new algorithm we devised in Section 7, and reiterate the verification approach. Since R/W LLAB is fine-grained and supports reader/writer locking, we are likely to obtain smaller blocking bounds and therefore tighter WCRTs and better schedulability. Furthermore, due to the low overheads of R/W LLAB (Section 8), tighter WCRTs will give us a higher confidence that our verification results will be preserved in practice, as the negligible overhead of R/W LLAB will likely not cause deadline misses when it adds to tasks’ WCRTs, in particular HRT tasks.

Listing 3:
Generating the largest WCET of TU codels per task (following the R/W LLAB protocol).

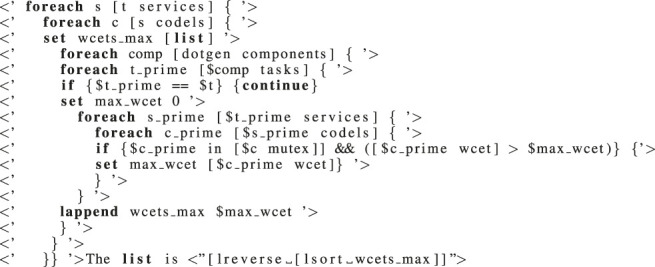

### 9.1 Step One: Schedulability Analysis

The only difference in this step compared to what we have seen in Section 4.1 is that the bounds *B*_*c*_ for each TU codel c are computed following the fine-grained reader–writer specificities of R/W LLAB instead of the single-memory nature of global MSRP. Therefore, a new algorithm to compute the bound *B*_*c*_ for a TU codel c in task *t* is summarized next. (1) For each task *t*′ ≠ *t*, we find, within all its services, the largest WCET of all TU codels **in conflict with** **c** (following the reader/writer conflict definition in Section 7). (2) We sort, in decreasing order, the values found in (1). (3) *B*_*c*_ is equal to the sum of the first |*C*| − 1 values sorted in (2). This gives the Tcl code in Listing 3. Contrary to Listing 1 (Section 4), here we need to compute *B*_*c*_ for each TU codel c in *t* separately because the set of codels in conflict with c may differ from one codel to another (loop starting in line 1). Then, the algorithm considers only the codels c’ in conflict with codel c in the computation (through the first literal of the conjunction in line 10) and ignores the rest. The rest of step one remains unchanged, where the new tighter bounds *B*_*c*_, computed using the new algorithm above, are used to compute tasks’ WCETs and WCRTs. One important remark is that this algorithm could not have been used with the original multi-resource reader/writer locking model of GenoM3 which, contrary to R/W LLAB, guarantees neither fairness nor starvation freedom (Section 6.1.3).

### 9.2 Step Two: Formal Verification

The only change in this step is that concurrency handling is managed through R/W LLAB encoding in the UPPAAL-SMC model. This is trivial in UPPAAL-SMC using a classical queue^14^. The remaining details are unchanged since we use the same scheduler as in Section 4.

### 9.3 Results

We apply the same two-step approach with the changes indicated above (to comply with the R/W LLAB implementation) to the same drone case study (Section 2.2). We consider the same affinity in Table 6, and the same properties are verified with the same statistical parameters for step two as in Section 5.

#### 9.3.1 Step One

The new WCETs of the five HRT tasks (resp., largest codel WCET in the three lower-priority tasks) in the system are given in Table 10 (resp., Table 11). The new WCRTs of HRT tasks are given in Table 12.

**TABLE 10:** New WCET of HRT tasks.

HRT task	WCET (ms)
main	0.32
comm	0.26
io	0.33
filter	0.29
control	0.42

**TABLE 11:** New WCET of largest codel in low-priority tasks.

Task	WCET of longest codel (ms)
publish	0.22
plan	0.19
exec	0.17

**TABLE 12:** New WCRT of HRT tasks considering affinity in Table 6.

HRT task	WCRT (ms)
main	0.58
comm	0.58
io	0.55
filter	0.46
control	0.59

Notice how each HRT task passes the schedulability test with a WCRT comfortably lower than its deadline. This is a direct effect of using a predictable fine-grain reader/writer locking protocol instead of a global one in this type of application, which confirms our observations in Section 8.2.

#### 9.3.2 Step Two

We generate, from the affinity in Table 6, the number of cores, and the GenoM3 system, an UPPAAL-SMC model where concurrency is handled using R/W LLAB. Then, we verify the same bounded response properties as in Section 5. The verification results are given in Table 13.

**TABLE 13:** New verification results (step two).

	*prob*_*t*_ ∈			
***t***	***x*_max_ = 2**	***x*_max_ = 3**	***x*_max_ = 4**	***x*_max_ = 5**
publish	[0, 0.004]	[0.996, 1]	[0.996, 1]	[0.996, 1]
plan	[0, 0.004]	[0.996, 1]	[0.996, 1]	[0.996, 1]
exec	[0, 0.004]	[0, 0.004]	[0.996, 1]	[0.996, 1]

Here, we notice that lower-priority tasks are most likely schedulable as well, with a *99*.*8%* probability as soon as *x*_*max* = 3 ms for tasks publish (period 4 ms) and plan (period 5 ms) and *x*_*max* = 4 ms for task exec (period 5 ms). Here also, the tighter bounds induced by R/W LLAB lead to a highly likely comfortable schedulability of lower-priority tasks, thus conforming, up to a high probability, with mission criticality as well.

### 9.4 Discussion

Using R/W LLAB allowed to improve the verification results in Section 5, where global MSRP was used instead. This improvement coincides with our expectations following the improved blocking bounds of R/W LLAB and our evaluation in Section 8. The new model, considering R/W LLAB instead of global MSRP, guarantees schedulability of all tasks, therefore complying with both safety and mission criticality, which was unverifiable in the original model (because of the scalability issues of the original GenoM3 system including an unpredictable locking model) and insufficient using global MSRP. The verified model does not take R/W LLAB implementation overheads into account (that is, we would have exactly the same results with lock-based R/W DGL). We argue, however, that the low overheads of R/W LLAB compared to other locks including R/W DGL, experimentally evaluated in Section 8, combined with the significant difference between tasks’ WCRTs and their deadlines (Section 9.3), would lead to the schedulability of all tasks preserved in the runtime setting.

However, it is possible, for other applications, that step one is not conclusive, that is, we fail to find an affinity that allows all HRT tasks to pass the schedulability tests. In this case, we may consider redesigning the application by, e.g., changing the periods, which is nevertheless not always feasible because periods may be dictated by hardware constraints (e.g., sensor frequency).

## 10 Related Work

In this section, we review the state-of-the-art on both verification of real-time robots (Section 10.1) and locking choices in real-time–oriented robotic frameworks (Section 10.2).

### 10.1 Rigorous Verification of Real-Time Robotic Applications

One of the main issues hindering the use of schedulability analysis is the generalization of tests to robotic task models (Gobillot et al., 2019). Some robotic software initiatives try to tackle this issue (Soetens and Bruyninckx, 2005; Schlegel et al., 2010; Gobillot et al., 2019). In particular, MAUVE (Gobillot et al., 2019) supports specification, implementation, and analysis of real-time constraints. Other works propose some real-time extensions for the popular framework ROS (Wei et al., 2016; Saito et al., 2018). However, all these works focus on adding schedulability features, sometimes with schedulability analysis support, and thus leave important properties such as reachability and bounded response unattended.

On the other hand, a major challenge of using formal verification is bridging robotic software, not formally founded, with formal methods. Proposed solutions range from ad hoc non-reusable formalization (Kim and Kang, 2005; Molnar and Veres, 2009) to formal frameworks for robotics (Miyazawa et al., 2017). Another difficulty is the lack of scalability of exhaustive verification techniques due to the complexity and size of robotic systems. Non-exhaustive techniques, such as SMC, used by Hazim et al. (2016), are not suitable for critical applications where schedulability of HRT tasks must be verified with certainty. Besides, to the best of our knowledge and except for our efforts (Section 10.1.1), the literature on formal verification in robotics (including works cited here) ignores the MEC and OS scheduling constraints, which restricts the results’ validity to the unrealistic assumption of all tasks running in parallel at all times.

#### 10.1.1 Our Previous Work

In Foughali (2017), Foughali (2019), and Foughali and Hladik, (2020), we proposed automated support to verify various properties of robotic applications under different scheduling policies by means of model checking. Such support is not suitable for the drone navigation application because of scalability issues. In Foughali et al. (2019b), we proposed an automated approach based on SMC to verify, up to a high probability, a number of properties. This approach is not suitable either for the drone system because SMC guarantees are not enough for critical properties such as the schedulability of HRT tasks. In other works, we propose the use of runtime verification (RV) to cope with the scalability issues of model checking (Foughali et al., 2020; Ocón et al., 2020). Though lightweight and scalable, RV techniques check properties as the system executes and are thus not suitable for critical applications where guarantees are needed prior to system deployment.

### 10.2 Real-Time Locking in Robotics

Besides ROS, there is a large corpus of robotic frameworks in the literature with different philosophies, capabilities, and design choices [e.g., YARP (Metta et al., 2006), OpenRT-M (Ando et al., 2005), and ArmarX (Vahrenkamp et al., 2015)]. In our state-of-the-art, we only focus on those frameworks that are real-time oriented, that is, developed for real-time applications, mainly ROS2, OROCOS (Bruyninckx, 2001), MAUVE (Gobillot et al., 2019), and GenoM3 (Mallet et al., 2010). As explained in Section 1.1, ROS2 is still under development and its real-time capabilities are yet to be understood (Casini et al., 2019; Blass et al., 2021; Choi et al., 2021). As seen in Section 10.1 above, MAUVE (Gobillot et al., 2019) is perhaps the most mature real-time–oriented robotic framework with full support for WCET estimation and schedulability analysis. Locking-wise, however, MAUVE relies on the OROCOS-RTT middleware (Section 2) where each component contains only one task, and resources are duplicated as *data flow ports* attached to components (Soetens and Bruyninckx, 2005). Then, whenever a component writes its own port, it diffuses the written value to components that need it by writing to their corresponding ports. This mechanism has two main disadvantages. First, it induces a memory constraint as most of data structures are duplicated. Second, access to ports is inaccurately referred to as “lock free.” Indeed, OROCOS-RTT still uses mutex procedures under the hood to, e.g., prevent a write attempt while a read is being performed (Santini and Lages, 2010), which makes it hard to reason on fairness (and therefore predictability) and efficiency. GenoM3 uses a low-level fine-grained, yet unpredictable concurrency model (Section 2.1.2). Perhaps, this lack of predictable and efficient locking mechanisms in real-time robotics is due to the fact that real-time locking is out of a roboticist’s expertise, but also the specificities of robotic software making it hard to apply state-of-the-art locks to real-time robots (Section 6.2). For instance, real-time–oriented robotic frameworks do not use nesting. They typically implement algorithms in “elementary codes” (*aka* codels, e.g., C functions as seen for GenoM3, Section 2) each requesting all resources for its execution (e.g., specified in its arguments) at once [codels are also used in MAUVE, see Gobillot et al. (2019), Section 3.1].

## 11 Conclusion and Outlook

In this work, we describe an automated two-step approach to rigorously verify complex (mixed-)critical real-time robots. It combines schedulability analysis and formal verification and is suitable for real-time robotic applications that do not scale with model checking. Our approach is automated for non-expert users and validated on a real drone case study. Furthermore, we present LLAB and R/W LLAB, two novel spin-based real-time locking implementations for multi-core real-time robots. They fulfill a set of requirements, based on real-time robot specificities, while outperforming the state-of-the-art multi-resource locks DGL and MRLock in predictability and/or efficiency. LLAB and R/W LLAB are also useful beyond the scope of robotics, i.e., in any multi-core/many-core real-time system with short critical sections that requires nesting-free multi-resource locking.

We give two examples of future work directions. First, though our experiments in Section 8 are carried out on a system that is inspired from a real robotic application, we still need to implement R/W LLAB in a robotic framework, e.g., GenoM3 or MAUVE, and evaluate its performance on actual executions of real-time robots. Second, our verification approach (Section 4, Section 9) does not include the locking-related overheads. While real-time analysis usually focuses on theoretical blocking bounds, recent works such as Nemitz et al. (2021) propose *overhead-aware schedulability analysis*. This will be a good starting point for us to quantify various overheads on real robotic implementations and include them in our verification process.

## Data Availability

The implementation of the locking protocols and the benchmarks are publicly available at https://gitlab.com/azuepke/llab/. The UPPAAL-SMC template is publicly available, since Foughali et al. (2019b), at https://github.com/Mo-F/uppaal-smc-exp.

## Footnotes

1
https://www.quanser.com/products/qdrone

2
https://docs.ros.org/en/rolling/Roadmap.html

3 “Resources” here, and throughout this paper, relate to memory and possible input/output devices and not to processor cores, the concurrency over which is handled through scheduling algorithms.

4 A number of references are provided in Section 1 where this claim was initially made. Following a consensus in both the robotic and real-time systems communities, this claim is the initial motivation of other articles such as Saito et al. (2018), referred to in Section 10.

5
https://www.openrobots.org/wiki/pocolibs

6 Actually, both MAUVE and GenoM3 support also the ROS-Com middleware but recommend using OROCOS-RTT and PocoLibs, respectively, for real-time applications.

7 In this case, there is an additional operation: *V* is assigned to the service status in *tab*_*t* to prevent *t* from executing it again at its next period.

8 Note how codels’ WCETs in Table 2 are much larger than the general trend of codels’ WCETs being short, less than 50 *μ*s, Section 2.2. This is because WCETs in Table 2 include the blocking bounds *B*_*c*_ as explained in Section 4.1.

9 A machine word is typically the largest unit in memory that can be changed atomically. The size of the machine word limits the number of resources.

10
https://gitlab.com/azuepke/llab/

11 A non-conflicting lock operation simply marks the requested resources as locked.

12 Read requests are accumulative. During an unlock operation, one cannot decide if *other* requests still have the same resources read-locked without checking all other requests in detail.

13 In MRLock, queue nodes are denoted as *cells* (Zhang et al., 2013).

14 Needless to use a lock-less queue here because timed automata provide a higher-level model where switching between states (*aka* taking transitions) is considered timeless, so blocking times do not include overheads unless the latter are specified explicitly in intermediate states.
